# Organoids in gastrointestinal diseases: from bench to clinic

**DOI:** 10.1002/mco2.574

**Published:** 2024-06-29

**Authors:** Qinying Wang, Fanying Guo, Qinyuan Zhang, TingTing Hu, YuTao Jin, Yongzhi Yang, Yanlei Ma

**Affiliations:** ^1^ Department of Colorectal Surgery Fudan University Shanghai Cancer Center Shanghai China; ^2^ Department of Cancer Institute Fudan University Shanghai Cancer Center Shanghai China; ^3^ Department of Oncology Shanghai Medical College Fudan University Shanghai China

**Keywords:** bench, clinical application, gastrointestinal diseases, organoids, scientific research

## Abstract

The etiology of gastrointestinal (GI) diseases is intricate and multifactorial, encompassing complex interactions between genetic predisposition and gut microbiota. The cell fate change, immune function regulation, and microenvironment composition in diseased tissues are governed by microorganisms and mutated genes either independently or through synergistic interactions. A comprehensive understanding of GI disease etiology is imperative for developing precise prevention and treatment strategies. However, the existing models used for studying the microenvironment in GI diseases—whether cancer cell lines or mouse models—exhibit significant limitations, which leads to the prosperity of organoids models. This review first describes the development history of organoids models, followed by a detailed demonstration of organoids application from bench to clinic. As for bench utilization, we present a layer‐by‐layer elucidation of organoid simulation on host–microbial interactions, as well as the application in molecular mechanism analysis. As for clinical adhibition, we provide a generalized interpretation of organoid application in GI disease simulation from inflammatory disorders to malignancy diseases, as well as in GI disease treatment including drug screening, immunotherapy, and microbial‐targeting and screening treatment. This review draws a comprehensive and systematical depiction of organoids models, providing a novel insight into the utilization of organoids models from bench to clinic.

## Graphical abstract

The established long‐term in vitro organoids models that have been derived from different gut segments, health status, ages, and species. Organoids models derived from different sources demonstrate distinct morphological and gene expression patterns.

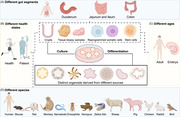

## 1 INTRODUCTION

Gastrointestinal (GI) diseases encompass a broad spectrum of disorders affecting the digestive system, including colorectal cancer (CRC), inflammatory bowel diseases (IBDs), and various functional GI disorders.^1^ The diagnosis of GI diseases typically entails a synthesis approach involving medical history estimate, laboratory experiments, physical examination, imaging studies (such as X‐rays or CT scans), and endoscopic procedures (such as colonoscopy or gastroscopy). At present, the treatment of GI diseases poses a multifaceted challenge due to the complexity of the digestive system, the diverse array of affecting conditions, and the varying responses of individual patients to therapeutic interventions. While medications such as anti‐inflammatory agents, immunosuppressants, proton pump inhibitors, and antibiotics can help control symptoms and prevent disease progression in many cases, they may not be effective for all patients or may cause adverse reactions. Surgical interventions, such as bowel resections, liver transplants, and tumor resections, carry risks of complications and require careful consideration of patient‐specific factors. In order to overcome those remaining challenges, we need to construct a deeper comprehension of the molecular mechanisms of GI diseases, which lies at the core of enhancing diagnosis and treatment.

The etiology of GI diseases is intricate and multifactorial.^2^ The prevailing consensus is that genetic factors play a critical role in disease progression as advancements in sequencing techniques over the past few decades have unveiled the mutations and aberrantly expressed genes associated with various states of GI diseases.^3^ However, targeted therapeutic strategies developed for these targets often encounter significant challenges during clinical trials, leading to unsatisfactory outcomes characterized by substantial side effects or trial failures.^4^ Moreover, success in the target development is not guarantee of positive response to treatment among all patients with corresponding diseases.^5^ This prompts us to reconsider our approaches as previous gene expression sequencing primarily directed toward the end state, rather than the origin state, of the disease. Therefore, further investigation into disease causality is imperative.

In addition to the genetic factors, environmental factors are believed to contribute to 85% of the etiological factors associated with GI diseases, with microbiota accounting for a substantial proportion of these environmental influences. Being considered as a “microbial organ,” the gut microbiome system is an important component of the gut microbes that include bacteria, fungi, bacteriophages, viruses, and protists.^6^ Numbered in trillions, they are densely distributed in the intestinal lumen and mucosa and are involved in physiological activities such as food decomposition, nutrient supply, and energy regulation in concert with the host.^7^ The dysbiosis of gut microbiome has been proved to trigger or exacerbate intestinal diseases, ranging from inflammatory disorders to malignancies.^8^, ^9^ In recent years, the employment of omics technologies and bio‐informatics, as well as the emerge of novel techniques such as high‐throughput sequencing and microbiota interactive modeling, have widely broaden our horizon of the relationship between gut microbiome and GI diseases.^10^, ^11^, ^12^, ^13^, ^14^ Stepping into the omics and postomics era, one of the forward‐looking considerations is how to adopt relevant models as reliable representations of the normal and diseased intestinal features to validate the associations between microbes and hosts found in big data analysis and to trace their correlation to causality with an eye to testing therapeutic targets.

However, the most widely used models in studying the molecular mechanism of GI diseases at present—cell lines and mouse models—exhibit significant limitations in both scientific investigations and clinical researches. It has been observed that many of the bench findings applying those models fail to be adapted for clinical settings. Mouse‐ or human‐derived cancer cell lines typically contain only one type of cells, thus it is likely to lose the heterogeneity of the primary tumor cells after several times of passages.^15^ As for mouse models, they are typically immunodeficient ones so that have great limitation in simulating the real immune response, not to mention the high cost of time consuming and resources during the disease modeling.^15^ Under these circumstances, organoids models bring out new insights into the biomedical studies. As a key technological overshoot, organoids models serve as an important link bridging the bench and the clinic. The development and application of organoids models hold great potential for enhancing our understanding of GI diseases as these models allow for the replication of complex tissue architectures and cellular interactions that are unable to be recapitulated in traditional two‐dimensional (2D) cell cultures. By culturing patient‐derived organoids, researchers can create personalized disease models that capture the unique genetic, molecular, and physiological characteristics of individual patients, shedding light on more targeted and effective therapies. In this event, the application of organoids models in both bench and clinic would undoubtedly be more prosperous in the near future.

The past few years have witnessed significant advancements in organoid‐related techniques, including different cultivation methods and strategies for enhancing cellular components to better emulate native organ structures.^16^, ^17^, ^18^, ^19^ These achievements have greatly contributed to the progress of biomedical research by enabling disease modeling, elucidating pathogenic mechanisms, and exploring therapeutic strategies. In order to get a comprehensive understanding of the current progression, it is crucial to summarize and analyze existing findings at the forefront of this field. Moreover, fostering the transition from basic research to clinical application is of paramount importance in ensuring the welfare of patients. In this review, we will make a broad and systematical introduction of the organoid application in both scientific researches and clinical studies. First, we will briefly describe an overview of the development history of organoids models. Next will be the bench utilization part, in which we will present a layer‐by‐layer elucidation of organoid simulation on host–microbial interactions, as well as the application in molecular mechanism analysis. As for the clinical application, we will provide a generalized interpretation of organoid application in GI disease simulation from inflammatory disorders to malignancy diseases, as well as in GI disease treatment including drug screening, immunotherapy and microbial‐targeting and screening treatment. Finally, we will make a prospective prediction of the future directions of organoids models in intestinal microecology, as growing trend toward utilizing sophisticated intestinal organoids and advanced techniques is a heated topic. It is our hope that this review will serve as a valuable reference for the advancement of organoids in biomedicine and clinical treatment, and foster interdisciplinary collaboration among professionals to facilitate the application of organoids in diagnosing and treating GI diseases.

## 2 OVERVIEW OF THE DEVELOPMENT HISTORY OF INTESTINAL ORGANOIDS MODELS

Organoids, also known as “mini‐organs,” refer to cells that are cultured in the three‐dimensional (3D) environment in vitro. They are capable of self‐organizing and differentiating into various kinds of functional cells.^20^ The first intestinal organoid model, derived from single Lgr5^+^ stem cells, was established by Hans Clevers team in 2009,^21^ marking the beginning of researches on the utilization of organoids models in various fields such as gut disease modeling, mechanism revealing, drug screening, clinical treatment testing and so on. Intestinal organoids were initially originated from the crypts of small intestine and then cultivated into a 3D structure, which simulated the primary intestinal epithelium.^22^ Intestinal organoids are mainly derived from three kinds of cells, either from embryonic stem cells, or induced pluripotent stem cells (iPSCs), or neonatal/adult stem cells. Stem cell‐derived organoids are induced to form microcell clusters and are cultured in an ex vivo 3D environment. Gut organoids derived from iPSCs in a stepwise manner could mimic embryonic development after embryo implantation under a complex coordinate specification, allowing us to determine the formation of the morphological features.^23^, ^24^ Multicellular compositions exist in the organoids models, including mucus‐producing goblet cells, absorptive enterocytes, chemosensory tuft cells, hormone‐secreting enteroendocrine cells, antimicrobial peptide‐producing Paneth cells, multipotent proliferative stem cells, and antigen‐sampling microfold cells. These cells share different functions in processes such as nutrient absorption, ion transport, mucus secretion, barrier protection, and microbicidal peptide production.^25^

**FIGURE 1:**
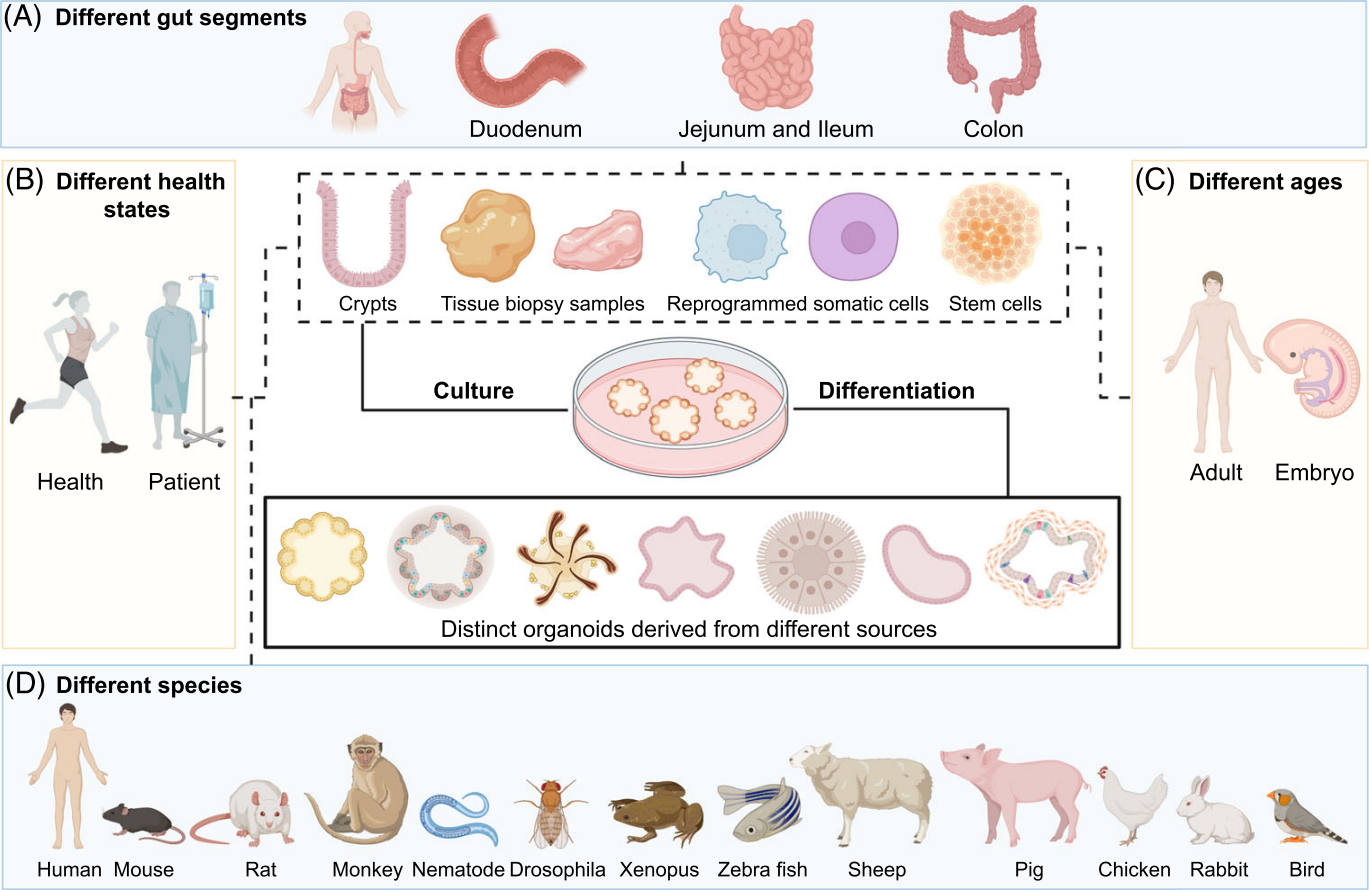
Schematic diagram summarizing the generated gut organoids varied in location, health status, age and species, location, and mode of differentiation. (A–D) The established long‐term in vitro organoids models that have been derived from different gut segments (A), health status (B), ages (C), and species (D). Organoids models derived from different sources demonstrate distinct morphological and gene expression patterns.

Nowadays, multiple organoids models have been developed and optimized in various directions with the endeavor of the scientific community all over the world. Not only from humans, organoids can also be generated from a broad range of other species, preserving and exhibiting some inherent characteristics of their origin. For instance, apart from human and mouse, organoids can be derived from other model organisms: rat, monkey, nemathelminth, drosophila, xenopus, zebra fish; from farm animals: sheep, pig, chicken; from wild animals: rabbit, bird, and so on.^26^, ^27^, ^28^, ^29^ Since the existing organoids models are able to take different gut segments (Figure 1A), health status (Figure 1B), ages (Figure 1C), and species (Figure 1D) into consideration,^24^, ^26^, ^30^, ^31^, ^32^, ^33^, ^34^ they are expected to facilitate the rapid transition from basic researches to clinical applications. Different segments of established organoids have variable transcriptome profiles and some pattern recognition receptors (PRRs), Toll‐like receptors (TLRs) 1, 2, and 6 for example, which are capable of showing strikingly species‐specific expression along the cephalocaudal axis.^34^ In addition to the position differences, adult organoids also show age differences as organoid cultures of early‐onset CRC reveal distinct and rare genetic profiles.^31^ Genes involved in maturation, gut barrier function, and innate immunity are different in fetal‐derived and adult‐derived intestinal organoids.^32^ Normal and disease‐derived organoids differ in factor secretion and gene expression as well as in growth dominance.^31^, ^33^ Meanwhile, except for the advances in modeling intestinal organoids, organoids models have also been established for other digestive tract organs, such as stomach,^35^ pancreas,^36^ esophagus,^37^ liver,^38^ and gallbladder.^39^ So far, a number of healthy and diseased GI tissues have been included in the organoids banks.^40^, ^41^ They faithfully recapitulate mutated genes, protein expression levels, and cellular composition observed in surgically resected primary tissues, providing an invaluable in vitro model for the investigation of digestive tract physiology and pathology.

## 3 ORGANOIDS MODELS IN BENCH: APPLICATION SCOPE OF GI ORGANOIDS MODELS

GI organoids have significantly deepened our understanding of the physiological functions within the GI tract over the past decade, showcasing their immense potential in disease simulation and therapeutic testing. For instance, specific gene knockouts can modulate the fate of stem cells,^42^ of which the certain molecules or signaling pathways play a crucial role in epithelial system differentiation.^43^ However, the epithelial organoids models of those studies have inherent limitations. In recent years, scientific researchers have been committed to the development of the coculture system of epithelial organoids to meet different research needs. For instance, coculture of epithelial organoids with immune cells can be used to construct immune microenvironments or conduct immunotherapy testing^44^, ^45^, ^46^; coculture with endothelial cells for vascularization investigation^47^, ^48^; coculture with neural cells to study the paracrine signaling effects.^49^ At present, both academia and some industries are focusing on how to optimize the cultivation system of organoids and develop multifunctional organoids models.

The GI tract can be broadly categorized into the mucosal layer, epithelial layer, immune layer, and vascular layer based on its physiological structure. These layers interact with each other in response to both endogenous and exogenous stimuli, such as microbial infections and organ function changes within the epithelial digestive tract. Currently, researchers are striving to integrating all these advancements to comprehensively simulate the structure and physiological activities of the entire GI tract. In this section, we utilize intestinal organoids as a model system to simulate microbial–host interactions and elucidate their impact on shaping the various structure and function of different intestinal barriers. Additionally, we explore the molecular and cellular responses triggered by microbial infections.

### 3.1 Application of organoids models in simulating the host–microbiota interaction

Recently, organoids models have been emerging in the study of gut microbes as they are capable of expressing the corresponding PRRs, such as NOD domain‐like receptors and TLRs. This feature allows them to be well tested in simulating the interactions between the microbes and intestinal epithelium, and even between certain specific cell types.^50^, ^51^ As a prominent in vitro model, organoids have overcome the substantial drawbacks of cell lines in simulating multiple action modes of microbiota on hosts, as well as the high cost of germ‐free mice and the insurmountable species differences between humans and animals.

The process of regulating intestinal diseases by microbiota and its byproducts is essentially a course in which microorganisms maintain or destroy intestinal homeostasis by regulating the integrity of the intestinal barrier. As the most complicated ecosystem in human body, the intestinal microorganisms consist of a variety of different species and are able to interact with the host cells through invasion or adhesion mode,^52^, ^53^ which can be further divided into the role played by the microorganisms themselves or by their derivatives such as virulence factors, proteins, or metabolites. Organoids models demonstrate the impact of microorganisms on the host through morphological changes, alteration in cell composition, and differences in gene expression. Being a powerful tool in bench, organoids models can be used to study the mechanism of the destruction or repair of gut barrier system at the macro level, while can also be used to study the regulation of gene expression, epigenetic inheritance, or cell composition at the micro level.

**FIGURE 2:**
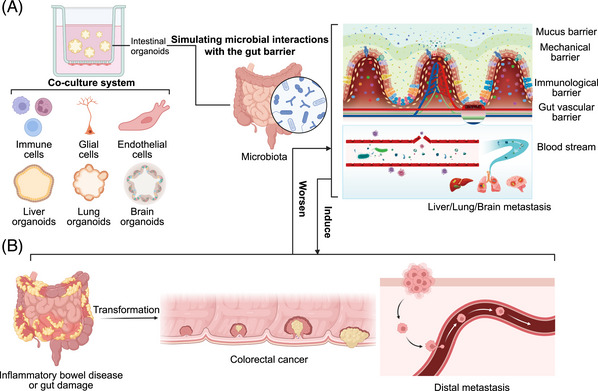
Organoids models simulating the process, mechanism, and results of the interaction between microbiota and intestinal barrier. (A) Organoids mimic the adhesion, invasion, immune invasion and distant metastasis of gut commensal bacteria or pathogens. The effects of microbial communities on the mucus barrier, mechanical barrier, immunological barrier, gut vascular barrier, and distant metastasis can be simulated by coculturing intestinal organoids with immune cells, glial cells, endothelial cells, and other organoids including liver, lung and brain. (B) The changes of various molecular cells mediated by microbiota infection affect the occurrence and development of intestinal diseases, such as inflammatory diseases and malignant tumors, which constantly form a feedback with intestinal microbiota and disease states.

The intestinal tract is an important organ with various diverse functions including digestion, nutrient absorption, and waste excretion.^54^, ^55^, ^56^ The two major components of the intestinal tract are small intestine and colon. The former one includes duodenum, jejunum, and ileum, in which the contained villil, microvilli, and crypts allow the small intestine to be the ideal place for nutrient absorption. The colon is composed of ascending, transverse, descending, and sigmoid colon, and is responsible for water, electrolytes, and vitamin absorption.^57^ The intestinal tract can be briefly divided into four layers of barriers against pathogen invasion. Luminal microbes and mucosa come from the mucus barrier; epithelial cells form the mechanical barrier; immune cells, Peyer's Patches (PPs) and intestinal lymphatic system form the immunological barrier; gut vascular barrier (GVB) is formed by endothelial cells, pericytes and enteric glial cells. By mixing or coculturing with stromal cells, or even with other organoids (such as brain, liver, and lung), intestinal organoids models could simulate various barrier structures or functions of the intestine, regardless of the presence of epithelial organs (Figure 2A). Through the observation of the interactions between various cells and microorganisms within these barriers, we can use organoids models to recapitulate the stress responses and innate or adaptive immune processes displayed by the gut, in order to combat intestinal diseases induced by bacterial infections and resist bacterial translocation or distal metastasis (Figure 2B). Here, we present the influence of microorganisms on intestinal barrier structure layer by layer in vivo to show the protective and destructive effects of microbiota on various intestinal barriers. Meanwhile, we demonstrate how could organoids models represent those effects through phenotypic changes. In addition, we also summarize the molecular mechanisms behind these interactions, which are presented as follows.

#### 3.1.1 The mucus barrier

The mucus of the large intestine consists of two layers. The first layer is the solid inner layer (or glycocalyx) associated with the epithelium. On the surface of this layer, mucins generally play a role in cleaning microorganisms and establishing protective barriers. The second layer is the sticky outer layer.^58^ These mucosal tissues contain a large amount of glycoproteins and immunoglobulins secreted by the lower epithelial cells. Pathogenic bacteria need to degrade and penetrate the mucus layer in order to colonize and infect the intestinal epithelium.^59^ Luminal and mucosal symbiotic microorganisms regulate the complex multimicrobial ecological network between the internal and external environment and fight against the invasion of pathogenic bacteria by promoting mucosal protein secretion, maintaining the integrity of barrier structure, and secreting protective factors.

Organoids are able to produce an intact mucus layer with thickness similar to that have been observed in the human colon, allowing for the colonization of various microbes.^60^, ^61^ Therefore, organoids models have significant advantages in detecting mucus formation, exploring competitive or synergistic effects between different microflora in the mucous layer and demonstrating how to use microflora to regulate the mucus thickness. The colonization of bacterial communities is conducive to the secretion of mucus and the maturation of mucus layer, which improves barrier function.^62^ Some probiotics use mucin as a carbon source to promote self‐colonization, so as to resist the colonization of harmful microorganisms, while others are able to release certain metabolites to increase the goblet cell number or differentiation capacity to hinder the invasion of pathogenic microorganism. Both mechanisms can be detected on the organoids models.^63^, ^64^, ^65^

#### 3.1.2 The mechanical barrier

Serving as a critical part of the gut barrier, the intestinal epithelium plays a critical role in the pathophysiology of both GI diseases and other nonintestinal diseases.^66^, ^67^, ^68^, ^69^ The gut epithelium consists of microvilli, tight junction (TJ) proteins, and a variety of epithelial cells with specific functions, forming a barrier system against the incursion of pathogenicity microorganisms.^51^ Moreover, the identification of microorganisms and the mobilization of downstream response elements can be realized through the recognition of receptors and precise regulatory patterns.^70^

By utilizing intestinal organoids, it can be explained that although the host expresses the same receptor, the subtle differences of flagellar structure in symbiotic bacteria and pathogenic bacteria may lead to the varying responses.^71^ Organoids with cellular heterogeneities, namely different entry receptor expression patterns, can be used to study the control of microorganisms on epithelial defense and regeneration.^61^, ^72^, ^73^ A number of microbial functions that reside in different functional niches of the intestine can regulate epithelial function, as well as recovery and repair. Organoids exhibit disruption of the epithelial system through parameters such as surface cell apoptosis, release of inflammatory factors, decreased expression of TJ proteins, and penetration of the epithelium. For instance, infection with the pathogenic bacterium *Salmonella typhimurium* or *Enterococcus faecium* leads to decreased expression of TJ and increased release of chemokines in organoids.^74^ In the enterogenic monolayer of human organoids containing M cells, the intestinal pathogen *Yersinia pseudotuberculosis* (*Yptb*) is observed to instigate specific M cell extrusion to enter and colonize PPs.^75^

Furthermore, intestinal homeostasis is maintained through a balance of orderly epithelial cell death and constant cell self‐renewal and differentiation based on the stem cells. Thus, functional damage of stem cells is another critical pathway through which some pathogenic bacteria may disrupt the intestinal mechanical barrier. Currently, differentiated organoids are coupled with numbers of stem cell components, which allows further researches of these potential functions. For example, *Listeria monocytogenes* infection will cause an increase in the secretion of proinflammatory cytokines, reduce the budding rate of organoids and further interfere with the differentiation of intestinal stem cells (ISCs).^76^, ^77^ *Clostridium difficile* binds to epithelial receptors through its virulence factor and disrupts the epithelial barrier structure by directly disintegrating the actin cytoskeleton or indirectly mediating stem cell dysfunction, thereby delaying epithelial repair time.^78^, ^79^

#### 3.1.3 The immunological barrier

The gut immunological barrier is thought to be another important defense line against the invasion of gut microbes. In response to the invasion of microorganisms, the gut has formed a complex immune defense network, containing a variety of immune cells corresponding to the innate and adaptive immune systems.^80^, ^81^ The intruding bacteria would be identified, phagocytosed and cleared by the mucosal innate immune cells composed of macrophages and dendritic cells (DCs) after passing through the mucosal and epithelial barriers. However, under pathological conditions, critically ill patients usually experience intestinal mucosal innate immune cell dysfunction, systemic immune deficiency or immunosuppression. Thus, these patients are leaving their immune barrier unable to eradicate invading pathogens, with the dysregulated microbiota in turn exacerbating immune system dysfunction. This situation leads to intestinal bacterial translocation and further mediates intestinal infection or tumor metastasis.

The coculture of organoids and immune cells can be used as a simple simulation of the intestinal immune barrier to evaluate the response of immune cells during microbial invasion. At present, some available coculture systems of organoids with lymphocytes or macrophages have been developed.^46^, ^82^, ^83^ Organoids models play an important part in many interesting examples of how microbial communities regulate communication and cooperation among barrier systems. In an organoid‐DCs coculture system, the metabolites secreted by *Lactobacillus reuteri* can mature DC cells and promote the production of IL‐10, thereby exerting anti‐inflammatory functions.^84^ Macrophages have a dual function of supporting organoids maturation and strengthening intestinal barrier. In the macrophage–enteroids coculture system, these macrophages can sense, capture, and kill intracellular enteropathogenic *Escherichia coli* through appendages across the enteroids monomolecular layer, without disrupting the epithelial barrier and inducing remarkable proinflammatory microenvironment.^85^ In addition, the regulation and composition of bacteria‐mediated microenvironment can also be observed in these kinds of coculture models. In colonic organoids cocultured with patient‐derived tumor‐infiltrating lymphocytes (TILs), exposure to *Fusobacterium nucleatum* increases the expression of PD‐L1 and the proportion of CD8^+^ and IFN‐γ^+^ CD8^+^ TILs in tumor organoids.^86^

Immune cells are also involved in organoids growth and can release factors into the microenvironment to regulate the stem cell function and organoids regeneration and maturation.^87^ The immune system plays a central role in tissue repair after clearance of infection. For instance, the innate lymphocytes (ILCs) of the lamina propria play a critical role in eradicating infection, alleviating inflammatory damage, and interacting with the overlying epithelium. They can also produce microbial‐derived signals that promote or hinder epithelial regeneration and are able to stimulate IL‐22 production by ILC3s.^88^ The capture of pathogenic microorganisms by immune cells with or without factors released by immune cells activates stem cells to repair the epithelial system. Likewise, organoids provide a great platform for modeling and studying the interaction between immune cells and epithelial cells in repairing damaged tissues after pathogen infection and tissue injury. *L. reuteri D8* promotes the growth of organoids and protects organoid morphology by increasing the number of LGR5‐expressing cells and accelerating the regeneration of ISCs upon tumor necrosis factor alpha (TNF‐α) treatment in the organoids and lamina propria lymphocytes coculture system.^89^ Valerate, a metabolite of gut microbiota, activates PGE2 production through PGE2^+^ macrophage subsets to stimulate Wnt/β‐catenin signaling pathway in ISCs by binding to EP1/EP4. This promotes self‐renewal of stem cells and organoids formation.^90^

#### 3.1.4 The GVB

The final intestinal barrier GVB, which consists of intestinal endothelial cells, associated pericytes and enteric glial cells, can actively prevent the spread of bacteria to other organs. Some bacteria can induce distal tumor metastasis by disrupting the GVB and forming a metastatic niche. For example, tumor‐resident *E. coli* disrupts the GVB through viral interferon regulatory factor and drives the spread of bacteria to the liver.^91^ These destructions further promote the formation of a niche conducive to cancer cell metastasis.

Organoids can be used to mimic distal metastases when cocultured with endothelial cells or enteric glial cells, or even with organoids derived from other organs.^47^, ^48^, ^92^, ^93^, ^94^ Since many coculture systems and fusion approaches are still under test, many current examples indicate this process by indirectly detecting other organ‐specific strains or damage to intestinal organoids. Chronic inflammatory liver diseases and ulcerative colitis (UC) are common complications of primary sclerosing cholangitis (PSC). Intestinal organoids infected with *Klebsiella pneumoniae* show PSC‐derived epithelial cells injury effects, which are associated with susceptibility to Th17‐mediated hepatobiliary injury and bacterial translocation.^95^ Exposure of Paneth cell‐depleted intestinal organoids to conditioned media derived from gut microbes significantly reduces tube formation and wound healing responses of endothelial cells.^96^ The production of Shiga toxins (Stxs) by enterohemorrhagic *E. coli* induces renal organoids damage and inflammatory response.^97^ Enterococcal family members containing enteroporins *Enterococcus* pore‐forming toxins (Epxs) can make intestinal organoids sensitive to Epx2 and Epx3 toxicity by binding human leukocyte antigen class I complex receptors and stimulating the expression of MHC‐I under interferon exposure. Epx2‐containing enterobacter faecium has a destructive impact on intestinal organoids and human peripheral blood mononuclear cells.^98^

Furthermore, multiorganoids models such as gut–liver, gut–lung, gut–brain, and even combined models are gradually being constructed. They are used to study the process of colorectal tumor metastasis to the liver, lung, and brain under microbial intervention, in order to elucidate the disease driving factors for distal metastasis. Considering that the various roles of gut microbes in enterogenic infections and distal metastasis of tumors are still under exploration, it is believed that the inclusion of multiorganoids models will provide a novel and powerful approach for studying disease development and mechanism resolution.

### 3.2 Application of intestinal organoids models in molecular mechanism analysis

**FIGURE 3:**
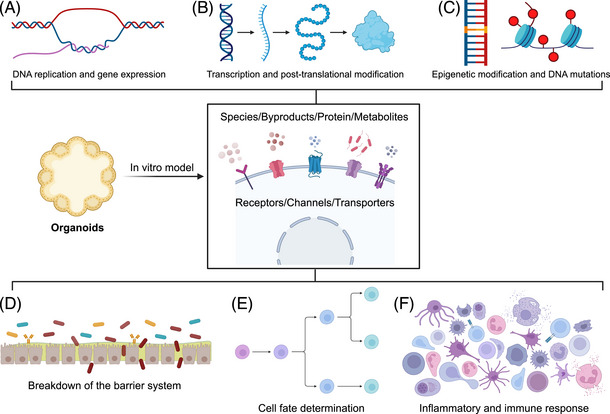
Organoids provide a versatile in vitro model for analyzing the molecular mechanism and biological functions of intestinal symbiotic bacteria or pathogens in intestinal infection. At the molecular level, microorganisms and their by‐products, such as proteins and metabolites, can be sensed by receptors, ion channels and transporters on the cell membrane to induce (A) genetic replication and gene expression; (B) the modulation of gene transcription and translation and posttranslational modification of proteins; (C) the regulation of gene epigenetic modification; (D) the disruption of the mucosal/epithelial/immune/endothelial barrier, allowing it to reach blood or lymph node and translocate to distant organs via the blood or lymphatic system; (E) cell fate changes manifested by proliferation, differentiation, transformation, and apoptosis; (F) the epithelium and immune cells that sense bacterial components via Toll‐like receptors, followed by the release of inflammatory cytokines and the regulation of immune cell recruitment/domestication.

The underlying cellular and molecular mechanisms have long been of great interest in the study of multiple interactions between hosts and microbiomes. In the past decade, organoids have provided a versatile in vitro model for the in‐depth and detailed research on the multiple mechanisms of microbiota and its metabolites on host, including epigenetic remodeling, DNA mutation, changes in gene expression and posttranslational modifications of proteins, cell fate determinations, disruption of the intestinal barriers, inflammation, immune activation, and so on (Figures 3A–F). Epigenetic mechanisms coordinate with healthy gut dynamics by sensing different microbial signals in response to changes in microbiota. The microbiota or microbial metabolites interfere with the host through epigenetic mechanisms and have long‐term effects.^70^, ^99^ A landmark study showed that *Muciniphila Akkermansia*, *Faecalibacterium prausnitzii*, and their short‐chain fatty acids (SCFAs) affect histone deacetylases and corresponding epigenetic modification in ileal organoids.^100^, ^101^ In addition, many pieces of evidence have shown that microbiota and its metabolites can mediate changes in physiological function by regulating the expression, activity, or modification level of epigenetic factors.^102^, ^103^

Stem cells have been proved to play an important role in the repair of the epithelial system. In certain cases of intestinal damage caused by external intrusion, stem cells can repair and fight against the loss of epithelial cells. The utilization of intestinal organoids models has also revealed the mechanisms through which intestinal microbiota directly or indirectly target stem cells to modulate the repair and epithelial cell regeneration during infection.^104^ Studies of intestinal organoids cultures exposed to SCFAs showed that those SCFAs had antiproliferative and prodifferentiation effects on ISCs through regulation of the transcription factor Foxo3.^105^ In addition to the stem cell epithelium, Paneth cells are also one type of cells that are often regulated by microbes. Emerging evidence has showed that western diet can convert primary‐to‐secondary bile acid (BA) conversion by commensal *Clostridium* species, such as deoxycholic acid, thereby altering the gut microbiome, activating farnesoid X receptor (FXR), and type I IFN pathways, and mediating Paneth cell defects, further affecting gut innate immunity and inflammation.^106^

In addition, gut microorganisms and their metabolites also participate in mutation accumulation, and regulate substantial plasticity of cancer mutations through the intestinal microenvironment. Human intestinal organoids chronically and repeatedly infected with pks^+^ *E. coli* showed the accumulation of mutation signatures caused by the bacterial genotoxin and those unique mutation signatures can be found in other independent cohorts.^107^, ^108^ Gut microbiome or its metabolites can switch mutated p53 cancer drivers in the small intestine back to normal p53 by regulating the tumor microenvironment, making it a “super repressor” that better inhibits cancer growth than the healthy p53 protein. Mutant p53 plays an antitumor role through the regulation of TCF4‐chromatin interactions and WNT activation, which is driven by gallic acid, a metabolite derived from the gut microbiota.^109^ This study highlights the role of the microbiota in determining of mutational plasticity in cancer.

Microbes and the corresponding metabolites also play an important role in immune regulation and inflammatory response. Lipopolysaccharide (LPS) exerts immunomodulatory effects by interacting with TLR4 on the cell surface. Additionally, it sensitizes multiple inflammatory signaling pathways by activating NF‐κB in organoids models.^34^ *Clostridium* strains selected from human stool samples modulate intestinal susceptibility and immune activity to IBD by inhibiting immune‐mediated NF‐κB activation in intestinal epithelial organoids derived from IBD patients.^110^

### 3.3 Application of intestinal organoids models in studying microbial composition changes in coordination with the host environment

**FIGURE 4:**
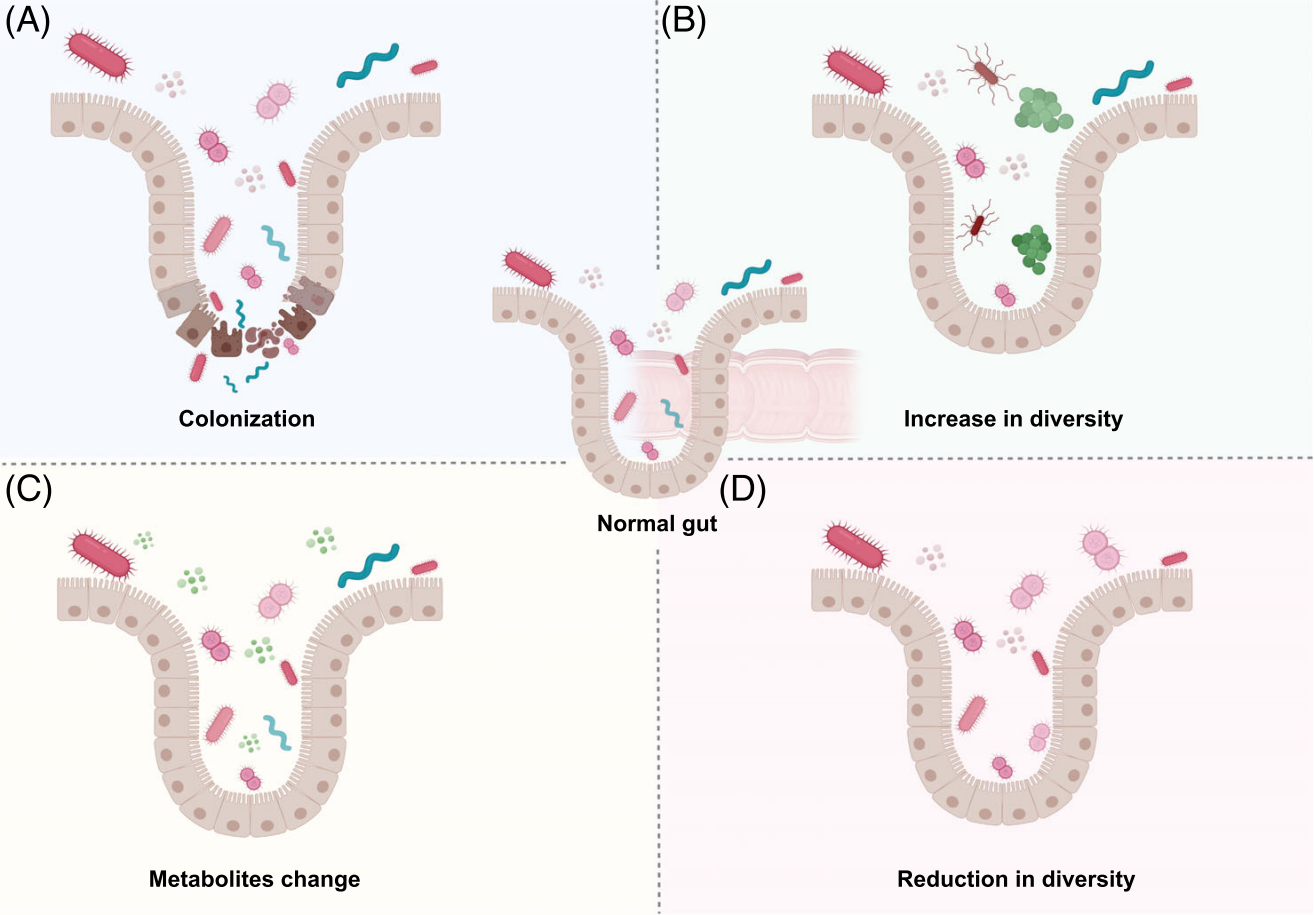
Microbial composition changes when external factors disrupt the harmonious operation. Metabolic molecules secreted or substances released due to gene expression, cell composition /fate change or death by the host will also react on the microbiota, presenting as (A) colonization, (B) increase or (D) reduction in diversity, and (C) the metabolic profile.

Microbial composition and host environment are in a coordinated relationship, and their interactions are mutual, even causally related. When external factors such as diseases, diet, drugs, competition among microbes themselves, genetic mutations, or deletions disrupt this harmonious pair, the composition of the microbes will correspondingly change, presenting as colonization (Figure 4A), changes in diversity (Figures 4B and D), and metabolic profile variation (Figure 4C). These conditions also have their own characteristics, specifically showing in the terms of bacterial colonization.

#### 3.3.1 Disease impact

Critically ill patients often have suppressed immune microenvironment, resulting in a dysregulated and persistent inflammatory circumstance that helps pathogens to maintain their own growth conditions. There are also many studies on the drug level. It is obvious that some antibiotics will undoubtedly affect the distribution of bacteria. In addition to antibiotics, microorganisms can also interfere with the therapeutic effect of many drugs by altering their bioavailability, and conversely, drug treatment can also affect the distribution and composition of microorganisms.^111^

#### 3.3.2 Lifestyle influence

It is also a common understanding that differences in lifestyle habits can lead to the variation of the microbial distribution in the body. Over the years, there have been some interesting studies, such as the influence of geography and family life on the composition of individual microbiota.^112^, ^113^ In recent years, many enlightening studies have been conducted on dietary habits, such as western diet, high‐fiber diet, ketogenic diet, and high‐fat diet, which can cause changes in the distribution characteristics of microbiota.^114^ It is for sure that these changes can respond at the molecular and cellular level, in other words, another perspective is what it brings to us at the micro level, regarding changes in gene expression or cell composition that alter the structure of microbiota. It has been shown that microbial infection‐induced cell death is usually a host defense mechanism that promotes the clearance of infection through the production of cell cadavers, such as pore‐induced intracellular traps, apoptotic bodies, and neutrophil extracellular traps.^115^ Gradually, there have been emerging researches on the complicated relationship between cell death and bacterial colonization. A recent study found that certain substances released after mammalian cell death can be used as nutrients absorbed by pathogenic *Salmonella* to induce the core transcriptional response of the pflB gene encoding pyruvate formate lyase, thereby helping bacteria colonize and proliferate in the gut.^116^

#### 3.3.3 Genetic mutations

Genetic factors also determine the composition of the gut microbiota. Multicohort big data samples showed that there was a correlation between human autosomal genetic variation and gut microbiome, and some polygenic structures may even determine the heritability of some gut bacteria.^117^ After the intervention of some disease‐related genes, changes in the composition and structure of some bacteria were detected. For instance, intestinal epithelial MHCII is a regulator of inflammatory responses to mucosal injury, T‐cell colitis, and intestinal infection. Furthermore, MHCII deletion reduces IgA binding to pathogenic bacteria and decreases bacterial clearance.^118^ Changes in the expression of some microbial sensors can also cause variation in the number of colonized bacteria.^119^

#### 3.3.4 Microbial competition

Bacteria influence the colonization and distribution of other bacteria through themselves and their metabolites. *Lactobacillus gallinatus* inhibits CRC progression by enriching probiotic abundance and depleting potential CRC pathogens.^120^ *Enterobacter faecalis* inhibits colonization and prevents *S. typhimurium* from dominating the microbiome.^74^ Succinate from the commensal microbiome drives gene expression and expansion in ATOH1‐independent tuft cells to combat eukaryotic colonization.^121^ The gut microbiota alters host cell function and differentiation in multiple ways to meet microbial needs.^122^ Taiotaomicron infection of organoids can directly induce the expression of fut2 in epithelial cells and enhance mucus to provide energy source for the bacteria.^123^ Compared with undifferentiated organoids, differentiated human colonic organoids are more likely to support the colonization and replication of *A. cmuciniphila* and *L. reuteri* or *Lactobacillus plantarum*, since they contain goblet cell‐derived mucins that act as bacterial carbon sources.^64^, ^124^ The colonization of microbiota under normal physiological conditions is the result of harmonious multicellular operations. Upon further consideration, these macro and micro levels may be cause‐and‐effect relationships. For example, the use of diet and drug can change the expression of certain genes and cell components, thus affecting the distribution differences of bacterial community.

At present, the correlation is not very systematic and comprehensive. At the micro level, it is more likely that the changes we observed are caused by specific interventions at the genetic and cellular level. The mutual causality between microbiota and host is similar to the fallacy of chicken‐and‐egg. As various debates still remain, further investigation and evidence is required. Nevertheless, they have provided us with a lot of useful and interesting information in their respective directions. We believe that with the deepening of research, these relationships will be analyzed in the future, forming a linear or correlated causal relationship, thereby helping us gain a more comprehensive and clear understanding of the influence and correlation of this interaction.

## 4 ORGANOIDS MODELS IN CLINIC: GI DISEASE SIMULATION AND TREATMENT

### 4.1 Application of organoids models in GI disease simulation

GI diseases can exhibit diverse characteristics based on multiple factors such as age, location, severity, molecular typing, and so on. A key advantage of organoids models lies in their ability to faithfully recapitulate the genetic and molecular features of GI disease tissues. Furthermore, certain organoids offer the unique capability to clone and simulate mutant genes that are challenging to be replicated in a 2D culture setting. For instance, CRC frequently exhibits chromosomal instability, which can manifest distinct subclonal and regional variations in severity. By cultivating organoids derived from patients with GI disorders, we can investigate the underlying genetic and molecular pathways driving disease pathology and progression. Here, we present a comprehensive analysis of organoids models simulating GI diseases associated with tumors, inflammatory diseases, virus‐ and parasite‐related infectious diseases, rare disorders, and others.

#### 4.1.1 GI cancers

GI cancers can arise from different cell types within the digestive tract, leading to diverse presentations, prognoses, and treatment approaches. Malignancies are one of the most important parts of GI diseases. With the rapid development of the culture measure of organoids models, now both surgical resection tumor tissues and tumor cells obtained in liquid samples such as ascites and peripheral blood can be applied to generate patient‐derived specific organoids.^41^, ^125^, ^126^, ^127^, ^128^ The great advantage of organoids models derived from clinical patients is the unique genomic and functional characteristics inherited from the original sample, which is quite distinct from other experimental models.^129^ In addition to mimicking primary tissue characteristics, the utilization of tumor organoids enables the investigation of how different gene deletions or mutations directly impact stem cell proliferation or tumor cell apoptosis to further regulate the tumor occurrence and development.^42^, ^130^, ^131^, ^132^, ^133^ Besides, organoids models are also employed to elucidate the interactions between different cellular components within the tumor microenvironment,^134^, ^135^, ^136^ which leads to an indirect impact on facilitating tumor progression.^137^, ^138^, ^139^ Organoids models also enable the detection of alterations in gene expression and epigenetic modifications during precancerous stages.^140^, ^141^

Furthermore, organoids can be utilized to observe the temporal progression of precancerous evolution. The information obtained from sequencing cancerous tissues may represent a terminal stage and is not necessarily indicative of causality. Longitudinal sampling limitations often restrict comprehensive observation of the entire disease progression. The application of organoids models fills the gap in this area by making it possible for us to observe how a single gene mutation induces precancerous lesions and initiates early tumorigenic events. For instance, introduction of TP53 deletion mutations into normal gastric organoids revealed that TP53 deletion gradually leads to aneuploidy over two years and subsequently dominates clonal expansion in subsequent cultures.^142^ Longitudinal single‐cell sequencing analysis demonstrated the progression of malignant transcriptional programs in gastric organoids lacking TP53, thereby illustrating that organoids models can effectively depict the entire process of precancerous lesions induced by single gene mutations.^142^

Additionally, tumor organoids offer a valuable tool for the investigation of microbial infection impact on tumor progression. For example, the pks^+^ *E. coli* is associated with the development of CRC in terms of genomic damage, which has been proved in organoids models. Organoids long‐term or short‐term infected with pks^+^ *E. coli* showed characteristic insertion and deletion mutations, impaired differentiation, and typical genomic instability respectively.^107^, ^108^, ^143^ In terms of tumor cell proliferation and apoptosis, the invasive *E. coli* expressing fia/fimH/htrA isolated from colonocytes is associated with tumorigenesis by promoting the proliferation of colonic organoids.^144^ *Helicobacter pylori*, which colonizes specifically in the gastric mucosa of over 50% of the population worldwide, has long been proved to increase the risk of niche‐specific diseases including adenocarcinoma.^145^ Nevertheless, the precise underlying mechanisms through which *H. pylori* increases the cancer risk has not been fully understood. One of the viewpoints is that *H. pylori* infection induces increased DNA damage in Apc truncation stem cells, thereby potentially exacerbating the tumor progression.^146^ Wroblewski et al.^145^ revealed that the localization and expression of claudin‐7 in epithelial cells was significantly changed by *H. pylori* through the application of gastroids, providing new insights into the molecular interplay between oncogenic pathogens and human epithelial cells.

In addition to the microbiota themselves, some of their metabolites or byproducts are also associated with GI diseases. BA can drive the activation of colonic mesenchymal stem cells through the BA–FXR axis, promote the growth of tumor characteristics, and accelerate the expression of colon cancer stem cell‐related markers in colonic organoids.^147^ Some outer membrane vesicles released by *F. nucleatum* can activate TLR4 and NF‐κB to stimulate proinflammatory signals in organoids with underlying colon cancer and gastroesophageal reflux disease.^148^

#### 4.1.2 Inflammatory diseases

GI inflammatory diseases present a diverse group of conditions that affect the digestive tract, causing inflammation, discomfort, and potential complications. Ranging from chronic IBD diseases like Crohn's disease (CD) and UC to conditions like diverticulitis and celiac disease, inflammatory diseases have a significant impact on individual's life quality and overall health. The establishment of a multitude of GI inflammatory organoids models has provided a novel platform for the identification of molecular characteristics associated with inflammatory diseases and the elucidation of underlying pathogenic mechanisms. Organoids models representing varying degrees of disease severity can be employed to investigate disease progression^149^ and facilitate the validation of distinct gene deletions or mutations in disease development.^130^, ^138^ While the exact causes of many GI inflammatory diseases remain unclear, factors such as genetics, immune system dysfunction, environmental triggers, and dietary habits are believed to play significant roles.

Organoids models also provide a platform for investigating how immune cells infiltrate the gut epithelium, interact with epithelial cells, and contribute to disease pathogenesis. Inflammatory responses in GI diseases involve dysregulated immune activation and cytokine production, leading to tissue damage and inflammation. By coculturing organoids with immune cells or microbial communities, researchers can recreate the inflammatory microenvironment of the GI tract and study the specific immune‐mediated responses in a controlled setting. Microbial imbalance is one of the pathogenesis involved in inflammatory diseases and organoids models can be used to simulate disease progression and status caused by complex microbial infections. The intricate interplay between the gut microbiota and the host mucosal immune system influences IBD. Organoids models could reproduce the disease progression with the microbes originating from IBD patients.^106^, ^150^ Organoids from healthy subjects cultured in fecal supernatants or LPS of patients with irritable bowel syndrome (IBS) can induce expression of unique colonic epithelial genes, reflecting the pathophysiology of the disease.^151^, ^152^, ^153^

#### 4.1.3 Virus‐ and parasite‐related infectious diseases

Scholars have done a lot of remarkable work on studying the infection of viruses in the intestines and the gut response to viruses by applying gut organoids. The noncultivatable pathogens in vitro, such as Human noroviruses (HuNoVs), can be cultured in intestinal organoids and combined with gene editing technology to search for key genes in inflammation and viral replication for HuNoVs.^154^, ^155^ During the COVID‐19 pandemic, some patients developed GI symptoms, and organoids models detected that the novel coronavirus could infect intestinal organoids, specifically bind with intestinal epithelial cells and destroy the integrity of the epithelial system. Organoids models can quickly respond to the analysis of disease mechanisms and the screening and validation of possible targets.^156^, ^157^ Human rotavirus (HRV) is a major cause of diarrhea‐related mortality in children under the age of 5 years worldwide, but the current studies are strictly limited due to the fact that HRV growth is strongly restricted in most cell lines and animal models.^158^ Saxena et al.^159^ made a breakthrough over this clinical issue by establishing a novel human intestinal enteroid, which has been proved to recapitulate the in vivo HRV infection features. In addition, some intestinal parasites such as *Cryptosporidium* infection will cause diarrhea and malnutrition in children. Organoids models can be used to mimic the natural ecological niche of the parasites and observe the life cycle of these parasites and host responses.^160^Therefore, organoids models have shown incomparable advantages in simulating the pathogenic process and characteristics of pathogenic microorganisms, and even in the testing and verification of drug targets.

#### 4.1.4 Rare GI diseases

Rare GI diseases present significant challenges for patients, healthcare providers, and researchers due to their low prevalence, diverse clinical manifestations, and limited therapeutic options. These conditions encompass a wide range of disorders affecting the GI tract, including rare genetic syndromes, congenital malformations, and uncommon inflammatory or neoplastic conditions. Modeling rare GI diseases is therefore critical for comprehending the pathophysiology, elucidating disease mechanisms, and developing targeted therapeutic interventions. In the context of rare GI diseases, organoids models offer several advantages over conventional cell culture systems and animal models as they play a pivotal role in replicating disease characteristics and elucidating pathogenic mechanisms.

Gastric neuroendocrine carcinomas are aggressive malignancies that have been under‐investigated due to their rarity and lack of disease models. Inspiringly, GEP‐NEN organoids faithfully recapitulate the pathohistological features and the functional phenotypes of the original tumors, providing a valuable tool for studying this disease.^161^ Additionally, these organoids can effectively replicate the drug response observed in patients.^162^ The incidence of small intestinal neuroendocrine tumor, although rare, has shown an increase over the past four decades. Genome‐wide association studies have recognized a new missense mutation in LGR5, namely “rs200138614, p.Cys712Phe,” that is associated with this disease. The established organoid model provides insights into its etiology by demonstrating that overexpression of LGR5 p.Cys712Phe disrupts R‐Spondin‐LGR5 signaling and promotes stem cell proliferation.^163^ Cronkhite‐Canada Syndrome (CCS) is an exceptionally unusual noninherited polyposis syndrome, affecting only one in every million individuals. The etiopathogenesis and optimal treatment for this syndrome remain elusive due to its rarity and the absence of suitable model systems. The organoid model demonstrates the aberrant local epithelial 5HT production and the dysregulated control of epithelial stem cell proliferation in a human organ affected by CCS, resulting in an increased quantity of enteroendocrine cells.^164^ In conclusion, organoid technology exhibits immense potential in modeling rare GI diseases and advancing our comprehension of disease pathophysiology.

#### 4.1.5 Other diseases

In addition to the above mentioned GI diseases, simulations of several other intestinal diseases have also been presented in a series of studies. Intestinal ischemia/reperfusion (I/R)‐induced ferroptosis of intestinal cells is accompanied by the changes in intestinal microbiota and its metabolites, such as capsiate (CAT), which can be simulated by the hypoxia/reoxygenation (H/R) model of ileal organoids.^165^ Interestingly, recent studies have found that organoids models can also be used to elucidate the role of circadian clocks in pathogenic responses to microbial infection, and to expound the role of microbial and circadian networks in regulating intestinal epithelial homeostasis. Enteroid organoids of mice and humans demonstrated a circadian‐dependent necrotic cell death in response to *C. difficile* toxin B through rhythmic expression.^166^ The C‐type lectin antimicrobial peptide REG3γ coordinates circadian oscillations with key ileal microorganisms in a bidirectional manner. High‐fat diet can lead to abnormal REG3γ expression in the host and impair the microbial oscillators of host metabolic homeostasis.^167^

Organoids models mimicking the intestinal diseases mediated by microbial infection and the corresponding mechanism analysis are concluded in Tables 1 and 2, classifying human and mouse models into two categories.

**TABLE 1:** Human organoids models that mimic intestinal diseases mediated by microbial infection and the mechanism analysis.

Microorganisms/metabolites	Organoids model type	Effects	Mechanisms	Diseases	References
Yptb	Human ileal enteroid	Yptb type three secretion system causes M cell extrusion from ileal monolayers, Yops impede M cell function	YopE affected the Rac1‐GAP activity	Intestinal infection	^75^
*Clostridium difficile* toxin A and toxin B	Human colonic organoids	Cell rounding	Disruption or reorganization of the cytoskeletal structure	Gastrointestinal infections, diarrhea, and enteritis	^78^, ^79^
*Enterococcus faecalis* (Epxs)	Human and mouse organoids	Induce death and damages of human peripheral blood mononuclear cells and intestinal organoids in a toxin‐dependent manner	IFN‐g sensitize intestinal organoids to Epx2 and Epx3, immune suppression and epithelial barrier disruption	Multidrug resistant infections or infections with high mortality or alcoholic liver disease	^98^
*Pks* ^+^ *E. coli*	Human intestinal organoids	DNA damage, ID‐*pks*/ SBS‐*pks* signatures pinpointing bacterial “fingerprints” in DNA alterations found in CRC cells, alters in copy number variations and other mutations	DNA alkylation and the induction of double‐strand breaks	CRC	^107^, ^108^
			Improper repair of colibactin‐induced cross‐links		
*Clostridium bolteae* (AHG0001 strain)	CD and UC patients‐derived organoids;	Suppress inflammatory responses and endoplasmic reticulum stress	Suppress both cytokine and LPS‐driven chemokine/cytokine expression	IBD	^110^
	Winnie mice organoids		Suppress NF‐κB activation		
*Lactobacillus* species and metabolites 1,3‐diaminopropane	Human intestinal organoids (generated from human pluripotent stem cells)	Sense intestinal iron levels and attenuate host iron absorption	Repress intestinal iron absorptive pathways via the inhibition of basal HIF‐2α function and promote cellular iron storage via the induction of ferritin expression	Iron‐related disorders	^122^
*Helicobacter pylori*	Human gastroids	Aberrantly alter a cancer‐associated tight junction protein	Alter the localization and expression of claudin‐7 in epithelial cells mediated by β‐catenin and snail activation	Gastric adenocarcinoma	^145^
*Fusobacterium nucleatum* (>50 kDa outer membrane vesicles)	Human colonoid monolayer	Stimulate secretion of the proinflammatory cytokines IL‐8 and TNF	Stimulate p‐ERK, p‐CREB, and NF‐κB activation	Intestinal inflammation and CRC	^148^
Concentrations of microbiota of UC patients	Human organoid (derived from UC patients and non‐IBD controls)	Dose‐dependent response of inflammatory markers and alterations in transepithelial electrical resistance measurements	Induce transcriptomic stress pathways including activation of EGR1, MAPK and JAK/STAT signaling, as well as AP‐1 family and FOSL transcripts	UC	^150^
Fecal supernatant or LPS (IBS patients or Gram‐negative bacteria)	Human colonoids and mouse colonoids	Distinct colonic epithelial gene expression	Activate mast cells to release PGE2 through the induction of the COX‐2 enzyme and to downregulate SERT expression	IBS	^151^, ^152^, ^153^
		Increased mucosal 5‐HT	Activite TLR4‐MyD88 signaling pathway		
		Downregulated Lox5 mRNA expression and synthesis of RvD1			
SARS‐CoV‐2	Human intestinal organoids	Induce stronger IFN response	SARS‐CoV‐2 infects enterocyte lineage cells with high ACE2 expression	Intestinal inflammation and damage	^156^
HRV	Human intestinal enteroid	Simulate human intestinal physiology and pathophysiology and epithelial response after HRV infection	Support robust replication of HRV G3P[8] and G1[P8]	HRV‐related diarrhea	^159^
*Clostridium difficile* (*Clostridium difficile* TcdB)	Human and mouse enteroids pluripotent stem cell‐derived human intestinal organoids	Circadian rhythms and circadian phase‐dependent necrotic cell death	Disruption of *Rac1* abolished clock‐dependent necrotic cell death	Rhythm‐related diseases	^166^
*Bifidobacterium longum*	Primary cultured enteroids	Promote the proliferation of organoids	Upregulation of the stem niche factors WNT3A and TGF‐β	IBS	^168^
*A. muciniphila BAA‐835*	Human and mouse organoids	Significantly larger organoids	Promote ISC proliferation in a Wnt3‐dependent manner	Radiation and chemotherapy gut damage	^169^
*Lactobacillus paracasei*	Human intestinal organoids (derivate from CD patients)	Prevent the inflammatory effects	Decrease NFkβ phosphorylation	CD	^170^

Abbreviations: Yptb, *Yersinia pseudotuberculosis*; GAP, GTPase‐activating protein; Epxs, enterococcus pore‐forming toxins; *E. coli*, *Escherichia coli*; ID‐*pks*, indel signature induced by *Pks* ^+^ *E. coli*; SBS‐*pks*, *pks*‐specific single base substitution signature; CRC, colorectal cancer; CD, Crohn's disease; UC, ulcerative colitis; LPS, lipopolysaccharide; IBD, inflammatory bowel disease; IL, interleukin; TNF, tumor necrosis factor; ERK, extracellular signal‐regulated kinase; AP‐1: activator protein‐1; IBS, irritable bowel syndrome; PGE2, prostaglandin E2; SERT, serotonin reuptake transporter; SARS‐CoV‐2, severe acute respiratory syndrome–coronavirus 2; IFN, interferon; ACE2, angiotensin converting enzyme 2; HRV, human rotavirus; ISC, intestinal stem cells; NFkβ, nuclear transcription factor‐B.

**TABLE 2:** Mouse organoids models that mimic intestinal diseases mediated by microbial infection and the mechanism analysis.

Microorganisms/metabolites	Organoids models	Effects	Mechanisms	Diseases	References
*Listeria monocytogenes*	Mouse organoids	Reduce budding rate and increase mortality of organoids	Interfere with the differentiation of ISCs	Systemic infections	^77^
*L. reuteri* ATTC PTA 6475	Mouse colonic organoids and DCs coculture system	Suppress inflammation	The metabolites and surface components promote DCs maturation and IL‐10 production	Acute colitis	^84^
Butyrate	Mouse organoids	Promote intestinal homeostasis	Repress HK2 expression via histone deacetylase 8 and reduce mitochondrial respiration	IBD	^103^
*Clostridium spp*	Mouse SI organoids (WT/Fxr^−/−^)	Inhibition of gut innate immunity and activation in gut inflammation	Increase the secondary BA deoxycholic acid levels and inhibit Paneth cell function	CD	^106^
Gallic acid	Mouse organoids (CKIa/p53 DKO/Apc^Min/Min^)	Promote normal growth and differentiation of tumor organoids	Disrupt the WNT pathway through preventing the binding of TCF4 to chromatin	CRC	^109^
Succinate	Mouse enteroids (Atoh1^−^/^−^)	Expanded tuft cells and reduced intestinal inflammation	Increase GATA3^+^ cells and type 2 cytokines (IL22, IL25, IL13), and decrease RORGT^+^ cells and type 17 cytokines (IL23) in a tuft cell‐dependent manner	CD	^121^
Invasive *E. coli* (fimA/fimH/htrA‐expressing)	Mouse organoid (developed from mouse colonic crypts)	Larger colonic organoids sizes, hyperproliferative, and tumorigenic abilities	Manipulate epithelial antimicrobial responses and suppress epithelial autophagy	CRC	^144^
BA (bile salt hydrolase‐encoding bacteria)	Mouse colonic organoids	Accelerate the expression of colon cancer stem cell‐related markers in colonic organoids	Activation of colonic mesenchymal stem cells through the BAs‐farnesoid X receptor axis	CRC	^147^
Capsiate (metabolite of the gut microbiota)	Mouse ileum organoids (hypoxia/reoxygenation model)	Reduce ferroptosis‐dependent intestinal I/R injury	Enhance Gpx4 expression and inhibit ferroptosis by activating TRPV1	Intestinal I/R injury	^165^
*Bifidobacterium longum*	Primary cultured enteroids	Promote the proliferation of organoids	Upregulation of the stem niche factors WNT3A and TGF‐β	IBS	^168^
Pravastatin	Mouse organoids	Attenuate intestinal I/R injury	Promote the release of IL‐13 from ILC2s via IL‐33/ST2 signaling	I/R injury	^171^
*Lactobacillus acidophilus*	Intestinal organoids (irradiated mice)	Enhance intestinal epithelial function	Induce sufficient differentiation in epithelial cells, particularly in mucin‐producing cells	Irradiation‐induced intestinal damage	^172^
*L. reuteri*	Mouse organoids	Protect intestinal organoids from *C. rodentium*‐induced damage	Activate the Wnt/β‐catenin pathway to avoid overactivation	Pathological injury or intestinal inflammation	^173^
		Inhibit *C. rodentium* colonization	Reduce TNF and IL‐1β secretion		
			Induce ISCs differentiation toward Paneth cells		
*Lactobacillus rhamnosus GG*	Mouse colonoid and enteroid	Induce cell proliferation	Activate JAK–STAT signaling pathway	Gut injury	^174^
BA	Mouse organoids	Activation of ISCs and regeneration of the intestinal epithelium	Activate the G protein‐coupled BA receptor 1	Colitis	^175^
Propionate (Clostridia)	Mouse organoids	Decreased colitis, increased proliferation, and promoted regeneration	Induce Reg3 expression via short‐chain fatty acids receptor signaling	Dextran‐sodium sulfate‐induced colitis	^176^
Lactate (*Bifidobacterium* and *Lactobacillus* spp)	Mouse organoids (Gpr81^−/−^)	Promoted proliferation of ISCs in SI organoids	Enhance expression of Wnt/β‐catenin pathway‐related genes	Radiation and chemotherapy gut damage	^177^

Abbreviations: ISCs, intestinal stem cells; *L. reuteri*, *Lactobacillus reuteri*; DCs, dendritic cells; IL, interleukin; HK, hexokinases; IBD, inflammatory bowel disease; SI, small intestinal; BA, bile acid; CD, Crohn's disease; CRC, colorectal cancer; *E. coli*, *Escherichia coli*; I/R, ischemia/reperfusion; TRPV1, transient receptor potential cation channel subfamily V member 1; IBS, irritable bowel syndrome; TNF, tumor necrosis factor.

### 4.2 Application of organoids models in GI disease treatment

Organoids models offer a robust platform for investigating the potential mechanisms of GI diseases and evaluating potential therapeutic interventions. By culturing organoids derived from patients’ tissues or stem cells, researchers can delve into disease‐specific cellular and molecular pathways, encompassing aberrant cell signaling, perturbed immune responses, and dysfunctional metabolic processes. These disease‐specific phenotypes enable the study of disease progression, screening of prospective therapeutics, as well as identification of novel drug targets and mechanisms of action for GI diseases in a clinically relevant context.

#### 4.2.1 Drug screening

Drug screening plays a pivotal role in pharmaceutical research and drug development, aiming to identify candidate compounds with therapeutic potential for various GI diseases. Traditionally, drug screening relies on 2D cell culture and animal models, which often fail to accurately replicate the complex physiology and pathophysiology of human tissues. With the rapid growth of high‐throughput 3D organoids screening, including seeding geometries and alternative plate design, organoids models play a generally important part in drug screening and discovery.^125^, ^178^ The establishment of the organoids banks enables high‐throughput drug screening, and most GI disease organoids models exhibit in vivo responsiveness to therapeutic drugs.^179^ Furthermore, organoids models are employed for evaluating repurposed drugs originally intended for other diseases and assessing their efficacy against novel diseases.^180^, ^181^ They also serve as a means to continuously identify new targets and design targeted therapy strategies.^182^, ^183^, ^184^, ^185^ Organoids models also facilitate the exploration of alternative treatment approaches for challenging drug targets. For instance, though certain therapeutic strategies targeting the WNT pathway often lead to adverse effects on normal crypt development, innovative approaches focusing on terminal differentiation have shown promise in resolving these issues. Furthermore, the establishment of organoids offers a valuable in vitro model for the continuous optimization of clinical treatment strategies, enabling extensive exploration of stratified and personalized therapies. For instance, esophageal‐gastric adenocarcinoma organoids were employed to investigate their response to combined 5‐FU and FLOT treatment, as well as their pathological correlation with patients. The threshold for stratified treatment could also be determined with the help of organoids models.^186^ Distinct mutation profiles call for tailored treatments, while different subtypes within the same disease require diverse therapeutic approaches.^131^, ^187^, ^188^ By combining disease staging and considering various stages and characteristics along with molecular classification, specific treatment strategies can be tested using orgnaoids models prior to clinical drug trials.^163^, ^189^, ^190^

Drug resistance has always been a major challenge in tumor therapy. Organoids models play a pivotal role in elucidating the mechanisms underlying drug resistance. These models enable the identification of drug‐resistant genes, cell populations, and even microenvironmental structures, thereby facilitating the design of targeted therapies based on these indicators. For instance, downregulation of drug resistance‐associated genes, induction of stem cell differentiation, and elimination of drug resistance‐promoting microenvironment were taken together, followed by a combination with chemoradiotherapy agents, to evaluate the synergistic effects on organoids models.^191^, ^192^, ^193^, ^194^, ^195^, ^196^, ^197^ Establishing organoids models at early stages of tumor progression helps to evaluate the effectiveness of specific disease‐targeted drug candidates and conduct molecular mechanism analyses. For example, novel drugs for serrated neoplasia pathway‐derived CRC can be screened after the culture of SSL patient‐derived organoids, and its specific gene expressions could be identified prior to tumor evolution. In recent years, significant advancements have been made in drug screening not only within organoids themselves but also through improvements in culture conditions and screening equipment such as high‐throughput culture devices for new drug screening.^198^, ^199^ Furthermore, organoid‐based drug screening enables the investigation of complex disease mechanisms and the identification of novel therapeutic targets. By studying the behavior of diseased organoids in response to candidate drugs, researchers can uncover underlying disease pathways and identify potential drug targets for the further investigation.

Organoids models also play an important role in clinical trials, with a primary focus on drug screening. These models can be divided into two categories according to different purposes: (1) as a preclinical model or a parallel control model for predicting disease progression or drug efficacy; (2) as a validation model or a method for exploring the mechanism of clinical outcomes. In terms of the first category, clinical trials for metastatic CRC,^200^ gastroenteropancreatic neuroendocrine carcinoma,^201^ and celiac disease^202^ using organoids as a guideline have been conducted and achieved considerable results. Preclinical experiments based on organoids will not cost much time of patients, so it will hardly delay the treatment process. Also, using organoids to screen out the most promising options can exclude ineffective treatment and improve the clinical outcomes of patients. As for the second category, drug tests designed for esophageal squamous cell cancer,^203^ CRC,^204^, ^205^ gastro‐oesophageal adenocarcinoma,^206^ IBD,^207^ and Lynch syndrome^208^ have also been on the way of conducting sub experiments based on patient‐derived organoids. Those sub experiments can verify the difference in the patients’ response to drugs and increase the credibility of trial results.

#### 4.2.2 Immunotherapy

Immunotherapy has emerged as a promising treatment approach for GI diseases, offering new avenues for targeted therapy and improved patient outcomes. Utilizing organoids models derived from patient samples has facilitated significant advancements in understanding and harnessing the immune system's role in GI disease pathogenesis and treatment. Organoids models allow researchers to study the interactions between immune cells, epithelial cells, and microbial components within the GI tract, providing insights into the complex immune responses underlying GI diseases. By culturing patient‐derived organoids, researchers can recreate the individualized immune microenvironment of GI diseases, enabling the investigation of immune dysregulation, inflammatory processes, and immune evasion mechanisms. In the context of immunotherapy, organoids models offer valuable tools for screening and optimizing immune‐based treatments for GI diseases. Researchers can assess the efficacy and safety of immunotherapeutic agents, such as chimeric antigen receptor T cells, immune checkpoint inhibitors, and adoptive T cell therapy, in organoids derived from patient tissues.^209^ This approach allows for the identification of personalized treatment strategies that target specific immune pathways or tumor antigens, thereby enhancing treatment efficacy and minimizing adverse effects.^210^

Furthermore, organoids models enable the study of immune‐mediated responses to GI diseases and therapeutic interventions.^211^ By coculturing organoids with immune cells, researchers can investigate immune cell infiltration, cytokine signaling, and immune‐mediated tissue damage in GI diseases, providing insights into disease progression and treatment responses.^212^ Additionally, organoids models allow for the evaluation of combination therapies that target both tumor cells and immune cells, potentially enhancing treatment outcomes and overcoming resistance mechanisms.^213^, ^214^ A microculture system was developed to meet the model building conditions for endoscopically resected small samples with limited tissue availability, and the feasibility of the treatment strategy was assessed within a 14‐day timeframe. T cell infiltration and immunosuppressant efficacy were evaluated by incorporating T cells and interstitial cells into an organoid model.^215^ In the future, these immune cells can be integrated into organoids to facilitate testing of diverse immunosuppressants, optimal timing and dosage for immunosuppressant treatment, identification of novel immunosuppressants, and enhancement of existing ones.

Despite the promising advancements, challenges remain in the development and utilization of organoids models for immunotherapy in GI diseases. Standardization of culture protocols, optimization of immune cell integration, and validation of findings in preclinical and clinical settings are among the key areas that require further research and development. Additionally, regulatory considerations and ethical implications surrounding the use of organoids models for immunotherapy warrant careful consideration and oversight. In conclusion, immunotherapy holds great promise for revolutionizing the treatment of GI diseases, with organoids models serving as invaluable tools for advancing research in this field. By leveraging the power of organoid technology alongside immunotherapeutic approaches, researchers can accelerate progress toward personalized and effective treatments for GI diseases, ultimately improving patients’ clinical outcomes and life quality.

#### 4.2.3 Microbial‐targeting and screening treatment

Meanwhile, under the microbial insight, organoids models also play a crucial role in the testing of microbial‐targeted therapeutic regimens and the screening of beneficial bacteria for the treatment of intestinal diseases. Some beneficial bacteria slow down the progression of intestinal diseases by modulating cell proliferation, apoptosis, immune responses, and microbiota composition. *L. gallinis* is one of the most depleted probiotics in the feces of CRC patients, which can eliminate tumorigenesis by promoting apoptosis of cancer cells in human CRC‐derived organoids.^120^ *Bifidobacterium longum* can upregulate Wnt3A and TGF‐β to promote organoids growth, suggesting a potential reason of alleviating IBS symptoms in the clinical setting.^168^ The organoids H/R model has been used as an alternative model for intestinal I/R injury. It was found that CAT or pravastatin, the intestinal microbiota metabolite, could attenuate H/R damage under H/R conditions.^165^, ^171^ Deficiency in budding and mucin expression have been observed in the intestinal organoids derived from irradiated mice; treatment with *Lactobacillus acidophilus* or its associated oligonucleotide 1 can promote post irradiation recovery, rescue the germinated organoids, and induce the full differentiation of mucin‐producing epithelial cells.^172^ In response to the intestinal injury induced by radiation and methotrexate, the strain *A. Muciniphila* AK32s show enhanced intestinal epithelial regeneration function, and are capable of maintaining ISCs stemness and repairing damaged intestine.^169^ Humanized *L. reuteri* is a probiotic that produces the antimicrobial compound reuterin, which can prevent *C. difficile* from colonizing the antibiotic‐treated fecal microbial communities and alleviate antibiotic resistance issues. Reuterin has been proved to have a protective effect against *C. difficile* toxin‐mediated cellular damage in human intestinal enteroid model.^216^

Some of the beneficial bacteria have protective effects of enhancing barriers, such as increasing TJ protein expression, reducing inflammation levels, and promoting stem cell function. *L. reuteri* can effectively repair gut damage after pathological injury by enhancing Lgr5^+^ stem cell amplification.^173^ *Lactobacillus rhamnosus GG* restored epithelial barrier functions after barrier disruption caused by proinflammatory cytokines by normalizing TJ protein expression.^174^ *Lactobacillus paracasei* was able to reduce constitutive inflammation in organoids derived from celiac patients with damaged intestinal mucosa.^170^

The metabolites, such as inositol‐1,4,5‐triphosphate, lactate, propionate, and BAs, can also induce the growth of intestinal organoids and promote epithelial repair.^99^, ^100^, ^175^, ^176^, ^177^, ^217^ Some bacteria produce SCFAs to combat the breakdown of the intestinal barrier and inflammatory response of organoids derived from patients with enteritis.^218^ These data suggest that certain metabolites have the potential to treat IBD or various intestinal injuries. Butyric acid produced by butyric bacteria can inhibit the adherent‐invasive *E. coli* pathobionts associated with CD, indicating that butyrate are capable of helping maintain epithelial mitochondrial form or function when challenged by *E. coli*‐LF82. Therefore, decreased abundance of butyrate‐producing bacteria in IBD would contribute to loss of epithelial mitochondrial and barrier functions, which could further trigger disease and/or exacerbate a low‐grade inflammation.^180^

Bacterial tumor therapy (BCT) has great application prospects in the treatment of solid tumors. *Salmonella enterica servovar attenuated typhimurium strain* (STm) has been widely used in mouse xenograft models and orthotopic xenograft models of BCT. In vitro cultured tumor organoids showed reduced tumor stemness markers after STm treatment.^219^ Bacteriophages therapy, as a highly targeted approach, can be used to selectively remove invasive pathogens from the gut and prevent disease progression. Phage therapy is another area that has been extensively explored. In organoids models, human intestinal enteroid monolayers infected with phage showed a strong adhesion and aggregation phenotype of bacteria to cells, and significantly reduced enteroaggregative *E. coli* on the surface of organoids.^220^ Fecal microbiota transplantation (FMT) is a hot field with insufficient research, achieving unexpected results in the treatment of certain diseases.^221^, ^222^ However, current FMT treatment is controversial and has limitations such as transient outcomes, pathogen transfer, and difficulties in storage and reproducibility. How to select the appropriate specific flora for transplantation based on the pathological characteristics of patients requires in‐depth and long‐term exploration. The development of relatively safe, durable and effective antibiotics or vaccine approaches is also a great direction. Moreover, organoids models also demonstrate great application prospects in the screening and the in vitro test of oral drug and vaccine delivery. In fact, since animal tests are no longer required before human drug trials by United States Food and Drug Administration since 2023, organoids models gradually demonstrate their integrated advantage of providing drug features and individual physiology at the same time.^129^, ^223^, ^224^ Several laboratories have suggested the possibility of using organoids to promote the delivery of nanoparticle‐based drugs to the intestinal inflammation sites of IBD.^225^, ^226^ For instance, Tong et al.^227^ applied intestinal organoid monolayers to test the transport effectiveness of the constructed oral drug delivery vehicles. Similarly, Davoudi et al.^223^ used intestinal organoids to investigate the influence of surface charge on the transport of 5ASA loaded PLGA nanoparticles, further validating that organoids models could be utilized as a proper in vitro model to simulate the function of intestinal epithelium and evaluate the efficacy of nanoparticle‐based drug delivery.

Recent advances in the understanding of the gut microbiome have also opened new avenues for the treatment of some GI diseases. The relationship between billions of bacteria living in the human gut and the health of GI tract is a rapidly evolving research area, with probiotics and FMT being explored as potential treatments for conditions like *C. difficile* infection and IBD. However, bacterial therapy is a trend that needs to be carefully controlled to ensure clear effects and mechanisms, and organoids models are expected to play a role in the above‐mentioned therapeutic approaches against intestinal diseases.^73^, ^228^ How to convert pathological and molecular characteristics of patients into read‐values and parameters of organoids requires further consideration. In conclusion, the use of organoids models in microbial‐targeting and screening treatment represents a transformative approach to pharmaceutical research, offering a more physiologically relevant platform for identifying novel therapeutics and advancing precision medicine. By leveraging the power of organoid technology alongside complementary approaches, researchers can accelerate the drug discovery process and improve the translation of promising candidates from the laboratory to the clinic.

## 5 CONCLUSION AND PROSPECTS

The organoids models in GI research allows for the replication of complex tissue structures and cellular interactions that cannot be recapitulated in traditional 2D cell cultures. By culturing patient‐derived organoids, researchers can create personalized disease models that capture the unique genetic, molecular, and physiological characteristics of individual patients, paving the way for more targeted and effective therapies. Moreover, organoids models enable high‐throughput drug screening and precise medicine approaches, allowing researchers to identify novel therapeutic agents and predict patient responses to treatment more accurately. By testing potential drugs in organoids derived from patient tissues, researchers can assess the drug efficacy, toxicity, and side effects in a more clinically relevant context, ultimately accelerating the translation of promising therapies from bench to clinic. Furthermore, organoids models enable researchers to explore how microbial dysbiosis contributes to disease development and progression. Dysregulation of the gut microbiota composition and function has been implicated in various GI disorders, including IBD, CRC, and IBS. Organoids models allow for the study of how specific microbial species or communities influence epithelial barrier function, immune activation, and inflammatory responses within the GI tract, providing insights into disease mechanisms and potential therapeutic targets. Also, organoids models facilitate the screening and optimization of microbiome‐based therapeutics for GI diseases. By culturing organoids with defined microbial communities or fecal samples from patients, researchers can assess the effects of probiotics, prebiotics, and FMT on GI epithelial integrity, immune modulation, and disease progression. This approach allows for the identification of microbiome‐based interventions that restore microbial homeostasis and ameliorate disease symptoms in GI disorders.

**FIGURE 5:**
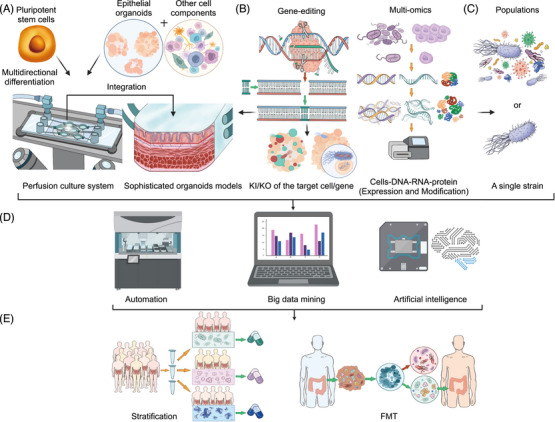
Future trends of microbial research on gut organoids. (A) More sophisticated organoids models, which more closely resemble the structure and function of the gut, can be developed in two ways, either by multipotent stem cell differentiation, or by integrating a variety of immune cells, mesenchymal cells, endothelial cells, and glial cells isolated from the gut. The resulting organoid cultured in a 3D‐printed perfusion system to monitor changes in culture factor and oxygen in real time. (B) The application of omics approaches in studying microbe–host interactions. Biological changes in strain‐host cell interactions have been studied to find and verify the protein targets of the interaction between microbiota and intestinal cells through multiomics and gene‐editing technology. This can be reached by knockout (KO) of the host or bacterial interaction proteins to verify the form of interaction between them, or by knockin (KI) of the target cell/gene into the green fluorescent protein, the strain/specific gene into the red fluorescent protein, or vice versa. Through this way, we can observe the specific interaction between them and analyze the physiological and functional changes (epigenetics, transcriptomics, proteomics, metabolomics, and single cell) of the two sides during the process of interaction. (C) Modeling the effects of the populations (a particular community) or single strains in organoids. (D) Through the automatic collection of organoid model flux tests combined with bioinformatics for big data mining and association analysis, artificial intelligence can be used for treatment plan prediction before biomedical technology used to verify the predicted data. (E) Therapies that target the microbiota and stratify the risk factors for patients with microbial infection, and identify beneficial bacteria for fecal microbiota transplantation (FMT).

However, despite the tremendous potential, organoids models also present several challenges that must be addressed to realize their full clinical utility. A major bottleneck remaining in the development of organoids models is that few existing organoids are capable of reproducing all the cell types, maturity level and overall function of the original organs.^129^ The fact that different cell types have varied proliferation rate, growth factor and oxygen exposure requirements is one of the leading challenges. Reproducibility of the organoids models generated by multicellular or multitissue has been witnessed a reduction due to this obstacle.^93^ Meanwhile, the prior isolation approach and cultural conditions specific to the GI organoids models remain to be further explored.^22^ Furthermore, current research on GI organoids remains predominantly in the preclinical stage. Challenges also remain in the development and utilization of organoids models for studying microbes in GI diseases. The current epithelial organoids and coculture system are mostly performed with just simple heterogeneity components. Therefore, it is possible to advance the organoid culture systems that are more similar to cellular conformation, biological, and mechanical functions of the native gut in vivo by combining materials science.^229^, ^230^ 3D bioprinting^231^ and microfluidic devices^232^ in the future (Figure 5A). Sophisticated organoids may be obtained by the multidirectional differentiation of pluripotent stem cells, or the integration of epithelial organoids and other cells of gut, which can be used to elucidate the causal relationship of host–microbe interactions. Moreover, they might be used to define how different microorganisms or patient‐specific microorganisms, even the whole microbiota of gut, regulate intestinal physiological processes and pathological progression (Figure 5B). The close‐knit integration with gene editing^233^, ^234^ and multiomics,^235^, ^236^ which will provide important insights into the mechanisms underlying microbial–host interactions, is also being expected (Figure 5C). These technologies will enable comprehensive and detailed detection of the modification or expression levels of various components, from DNA to RNA to protein, so as to clarify the functional changes after the interaction between intestinal cells and bacteria. Subsequently, organoids combined with gene‐editing technologies, through the knock‐out or knock‐in of organoids or microbial target genes, is able to reveal protein molecules involved in microbe–host interactions, verify interaction patterns, and assess microbe‐induced functional changes. This allows us to focus on the development and validation of therapeutic targets, identification of specific cell populations of host involved in microbes recognition and interaction through combination with single‐cell techniques.^121^, ^237^ The complexity of the interwoven relationship between microbes and hosts is complicated by differences in each individual's genetic predisposition and microbial composition. Thus, it would be best to refine or even personalize the treatment of the microbiome. Also, it is necessary to develop automated facilities for large‐scale sample testing and data collection^215^, ^238^ and perform detailed correlation analysis with the help of continuously improved bioinformatics and big data. Making predictions with the help of AI technology,^239^, ^240^ and carefully verifying the conclusions are also critical to reveal correlations between microbial profiles and individual genetic profiles as well as among various treatment regimens (Figure 5D). The acquisition of these data will certainly benefit the treatment of patients with microbial infections, the risk stratification of patients,^241^ the screening of safe and specific antibiotics and other drugs, the identification of profitable components for FMT, the testing of vaccine development and phage therapy (Figure 5E). The combination and application of organoids models with these advanced technologies are expected to contribute to the future of personalized therapy.

In order to facilitate the future application of organoids models in personalized medicine, multidimensional efforts must be taken by multiple sectors of the society. Clinicians should collect diverse samples during surgery to generate patient‐specific organoids and gather comprehensive clinical information encompassing patients’ medical history, treatment methods, and even lifestyles. By leveraging big data analysis, genetic changes and cell composition of each sample can be detected to establish interactive datasets reflecting the correlation between diseases and their underlying causes. Researchers need to focus on optimizing the culture system of organoids, exploring models that closely resemble human body structure and function, deciphering developmental cues, as well as analyzing physiological and pathological mechanisms. Additionally, there has been a noticeable emergence of scientific research companies within the industry aiming to advance organoid establishment techniques along with drug screening capabilities for high throughput applications and testing novel immunotherapy methods. For clinical experimentalists utilizing micro‐organoids models established through endoscopic technology, these models serve as valuable tools for assessing drug responses in patients during clinical experiments and enabling prediction and enhancement of drug efficacy. Regulatory authorities play a crucial role in overseeing organoid development regulations alongside formulating ethical laws, with regulatory agencies assessing the safety, quality, and efficacy of GI organoids through preclinical studies, clinical trials, and manufacturing processes. Organoid‐based therapies and products should meet established standards for patient safety and efficacy before being approved for clinical use or commercialization. Ultimately, it is imperative to effectively coordinate the interaction among all stakeholders in order to foster a constructive and collaborative environment and promote progress of this field. In summary, the development and utilization of organoids models hold great promise for advancing our understanding of GI diseases and developing more personalized and effective treatment strategies. By addressing the challenges associated with organoids culture and integrating organoid‐based research with other experimental and clinical approaches, we can accelerate progress toward improving outcomes for patients with GI disorders.

To sum up, GI organoids, whether derived from PSCs or adult tissue, can be used as an accurate and reliable in vitro models to faithfully reflect the in vivo physiology. Organoids models boast the strong potential of providing new insights into GI disease and revealing the possible underlying mechanisms. Meanwhile, they also play an important role in helping develop therapeutic regimens for GI disease therapy. However, though organoids models are serving as an indispensable tool nowadays, it has to be admitted that the current organoids technology is still imperfect. Further quality and quantity improvements, as well as a more accurate cellular composition of stem cell‐derived tissues, are required for progress to continue apace. It is with no doubt that together with the continuous development of advanced technologies, organoids models will further unravel the effect and the underlying mechanisms of GI disorders. Integration of organoids and new technologies will provide new insights into the mechanism beneath physiological functions and pathological processes. Additionally, subsequent specific treatments cast light on new therapeutic approaches.

## AUTHOR CONTRIBUTIONS

Q. W. and F. G. prepared the figures. Q. W., F. G., T. H., Y. J., and Y. M. drafted the manuscript. Q. W., F. G., Q. Z., Y. Y., T. H., and Y. M. edited and revised the manuscript. Q. W., F. G., Q. Z., Y. Y., T. H., Y. J., and Y. M. approved the final version of manuscript.

## CONFLICT OF INTEREST STATEMENT

The authors declare no conflict of interest.

## ETHICS STATEMENT

Not applicable.

## Data Availability

Not applicable.

## References

[1] Sbeit W , Kadah A , Mahamid M , Mari A , Khoury T . The interplay between gastrointestinal and cardiovascular diseases: a narrative review focusing on the clinical perspective. Eur J Gastroenterol Hepatol. 2021;32(2):132‐139. 32516176 10.1097/MEG.0000000000001779

[2] Swanton C , Bernard E , Abbosh C , et al. Embracing cancer complexity: hallmarks of systemic disease. Cell. 2024;187(7):1589‐1616. 38552609 10.1016/j.cell.2024.02.009 PMC12077170

[3] Wahl RL . The interaction of genomics, molecular imaging, and therapy in gastrointestinal tumors. Semin Nucl Med. 2020;50(5):471‐483. 32768010 10.1053/j.semnuclmed.2020.06.002

[4] Sunkar S , Neeharaika D , Nellore J , Nachiyar VC , Peela S . Small‐molecule‐targeted therapies for gastrointestinal cancer: successes and failures. Crit Rev Oncog. 2020;25(4):311‐333. 33639060 10.1615/CritRevOncog.2020036206

[5] Frank MH , Wilson BJ , Gold JS , Frank NY . Clinical implications of colorectal cancer stem cells in the age of single‐cell omics and targeted therapies. Gastroenterology. 2021;160(6):1947‐1960. 33617889 10.1053/j.gastro.2020.12.080 PMC8215897

[6] Laterza L , Rizzatti G , Gaetani E , Chiusolo P , Gasbarrini A . The gut microbiota and immune system relationship in human graft‐versus‐host disease. Mediterr J Hematol Infect Dis. 2016;8(1):e2016025. 27158438 10.4084/MJHID.2016.025 PMC4848019

[7] Hillman ET , Lu H , Yao T , Nakatsu CH . Microbial ecology along the gastrointestinal tract. Microbes Environ. 2017;32(4):300‐313. 29129876 10.1264/jsme2.ME17017 PMC5745014

[8] Wolter M , Grant ET , Boudaud M , et al. Leveraging diet to engineer the gut microbiome. Nat Rev Gastroenterol Hepatol. 2021;18(12):885‐902. 34580480 10.1038/s41575-021-00512-7

[9] Cheng WY , Wu CY , Yu J . The role of gut microbiota in cancer treatment: friend or foe? Gut. 2020;69(10):1867‐1876. 32759302 10.1136/gutjnl-2020-321153 PMC7497589

[10] Lloyd‐Price J , Arze C , Ananthakrishnan AN , et al. Multi‐omics of the gut microbial ecosystem in inflammatory bowel diseases. Nature. 2019;569(7758):655‐662. 31142855 10.1038/s41586-019-1237-9 PMC6650278

[11] Mills RH , Dulai PS , Vazquez‐Baeza Y , et al. Multi‐omics analyses of the ulcerative colitis gut microbiome link Bacteroides vulgatus proteases with disease severity. Nat Microbiol. 2022;7(2):262‐276. 35087228 10.1038/s41564-021-01050-3 PMC8852248

[12] Mihindukulasuriya KA , Mars RAT , Johnson AJ , et al. Multi‐omics analyses show disease, diet, and transcriptome interactions with the virome. Gastroenterology. 2021;161(4):1194‐1207. e8. 34245762 10.1053/j.gastro.2021.06.077 PMC8463486

[13] Yachida S , Mizutani S , Shiroma H , et al. Metagenomic and metabolomic analyses reveal distinct stage‐specific phenotypes of the gut microbiota in colorectal cancer. Nat Med. 2019;25(6):968‐976. 31171880 10.1038/s41591-019-0458-7

[14] Kong C , Liang L , Liu G , et al. Integrated metagenomic and metabolomic analysis reveals distinct gut‐microbiome‐derived phenotypes in early‐onset colorectal cancer. Gut. 2023;72(6):1129‐1142. 35953094 10.1136/gutjnl-2022-327156

[15] Yuan J , Li X , Yu S . Cancer organoid co‐culture model system: novel approach to guide precision medicine. Front Immunol. 2022;13:1061388. 36713421 10.3389/fimmu.2022.1061388 PMC9877297

[16] Saorin G , Caligiuri I , Rizzolio F . Microfluidic organoids‐on‐a‐chip: the future of human models. Semin Cell Dev Biol. 2023;144:41‐54. 36241560 10.1016/j.semcdb.2022.10.001

[17] LeSavage BL , Suhar RA , Broguiere N , Lutolf MP , Heilshorn SC . Next‐generation cancer organoids. Nat Mater. 2022;21(2):143‐159. 34385685 10.1038/s41563-021-01057-5 PMC12276900

[18] Carvalho MR , Yan LP , Li B , et al. Gastrointestinal organs and organoids‐on‐a‐chip: advances and translation into the clinics. Biofabrication. 2023;15(4):42004. 10.1088/1758-5090/acf8fb 37699408

[19] Dao V , Yuki K , Lo YH , Nakano M , Kuo CJ . Immune organoids: from tumor modeling to precision oncology. Trends Cancer. 2022;8(10):870‐880. 35773148 10.1016/j.trecan.2022.06.001 PMC9704769

[20] Corro C , Novellasdemunt L , Li VSW . A brief history of organoids. Am J Physiol Cell Physiol. 2020;319(1):C151‐C165. 32459504 10.1152/ajpcell.00120.2020 PMC7468890

[21] Sato T , Vries RG , Snippert HJ , et al. Single Lgr5 stem cells build crypt‐villus structures in vitro without a mesenchymal niche. Nature. 2009;459(7244):262‐265. 19329995 10.1038/nature07935

[22] Davoudi Z , Atherly T , Borcherding DC , et al. Study transportation of drugs within newly established murine colon organoid systems. Adv Biol (Weinh). 2023;7(12):e2300103. 37607116 10.1002/adbi.202300103 PMC10840714

[23] Spence JR , Mayhew CN , Rankin SA , et al. Directed differentiation of human pluripotent stem cells into intestinal tissue in vitro. Nature. 2011;470(7332):105‐109. 21151107 10.1038/nature09691 PMC3033971

[24] Munera JO , Sundaram N , Rankin SA , et al. Differentiation of human pluripotent stem cells into colonic organoids via transient activation of BMP signaling. Cell Stem Cell. 2017;21(1):51‐64. e6. 28648364 10.1016/j.stem.2017.05.020 PMC5531599

[25] Beumer J , Clevers H . Cell fate specification and differentiation in the adult mammalian intestine. Nat Rev Mol Cell Biol. 2021;22(1):39‐53. 32958874 10.1038/s41580-020-0278-0

[26] Beaumont M , Blanc F , Cherbuy C , et al. Intestinal organoids in farm animals. Vet Res. 2021;52(1):33. 33632315 10.1186/s13567-021-00909-x PMC7905770

[27] Duque‐Correa MA , Goulding D , Rodgers FH , et al. Defining the early stages of intestinal colonisation by whipworms. Nat Commun. 2022;13(1):1725. 35365634 10.1038/s41467-022-29334-0 PMC8976045

[28] Zhang M , Lv L , Cai H , et al. Long‐term expansion of porcine intestinal organoids serves as an in vitro model for swine enteric coronavirus infection. Front Microbiol. 2022;13:865336. 35369438 10.3389/fmicb.2022.865336 PMC8967161

[29] Gabriel V , Zdyrski C , Sahoo DK , et al. Canine intestinal organoids in a dual‐chamber permeable support system. J Vis Exp. 2022;181;e63612. 10.3791/63612 35311824

[30] Qu M , Xiong L , Lyu Y , et al. Establishment of intestinal organoid cultures modeling injury‐associated epithelial regeneration. Cell Res. 2021;31(3):259‐271. 33420425 10.1038/s41422-020-00453-x PMC8027647

[31] Yan HHN , Siu HC , Ho SL , et al. Organoid cultures of early‐onset colorectal cancers reveal distinct and rare genetic profiles. Gut. 2020;69(12):2165‐2179. 32217638 10.1136/gutjnl-2019-320019

[32] Senger S , Ingano L , Freire R , et al. Human fetal‐derived enterospheres provide insights on intestinal development and a novel model to study necrotizing enterocolitis (NEC). Cell Mol Gastroenterol Hepatol. 2018;5(4):549‐568. 29930978 10.1016/j.jcmgh.2018.01.014 PMC6009798

[33] van Neerven SM , de Groot NE , Nijman LE , et al. Apc‐mutant cells act as supercompetitors in intestinal tumour initiation. Nature. 2021;594(7863):436‐441. 34079128 10.1038/s41586-021-03558-4

[34] Kayisoglu O , Weiss F , Niklas C , et al. Location‐specific cell identity rather than exposure to GI microbiota defines many innate immune signalling cascades in the gut epithelium. Gut. 2021;70(4):687‐697. 32571970 10.1136/gutjnl-2019-319919 PMC7948175

[35] Barker N , Huch M , Kujala P , et al. Lgr5(+ve) stem cells drive self‐renewal in the stomach and build long‐lived gastric units in vitro. Cell Stem Cell. 2010;6(1):25‐36. 20085740 10.1016/j.stem.2009.11.013

[36] Huch M , Bonfanti P , Boj SF , et al. Unlimited in vitro expansion of adult bi‐potent pancreas progenitors through the Lgr5/R‐spondin axis. EMBO J. 2013;32(20):2708‐2721. 24045232 10.1038/emboj.2013.204 PMC3801438

[37] DeWard AD , Cramer J , Lagasse E . Cellular heterogeneity in the mouse esophagus implicates the presence of a nonquiescent epithelial stem cell population. Cell Rep. 2014;9(2):701‐711. 25373907 10.1016/j.celrep.2014.09.027 PMC4223874

[38] Miyajima A , Tanaka M , Itoh T . Stem/progenitor cells in liver development, homeostasis, regeneration, and reprogramming. Cell Stem Cell. 2014;14(5):561‐574. 24792114 10.1016/j.stem.2014.04.010

[39] Lugli N , Kamileri I , Keogh A , et al. R‐spondin 1 and noggin facilitate expansion of resident stem cells from non‐damaged gallbladders. EMBO Rep. 2016;17(5):769‐779. 26993089 10.15252/embr.201642169 PMC5341509

[40] van de Wetering M , Francies HE , Francis JM , et al. Prospective derivation of a living organoid biobank of colorectal cancer patients. Cell. 2015;161(4):933‐945. 25957691 10.1016/j.cell.2015.03.053 PMC6428276

[41] Vlachogiannis G , Hedayat S , Vatsiou A , et al. Patient‐derived organoids model treatment response of metastatic gastrointestinal cancers. Science. 2018;359(6378):920‐926. 29472484 10.1126/science.aao2774 PMC6112415

[42] Luissint AC , Fan S , Nishio H , et al. CXADR‐like membrane protein regulates colonic epithelial cell proliferation and prevents tumor growth. Gastroenterology. 2024;166(1):103‐116. e9. 37716376 10.1053/j.gastro.2023.09.012

[43] Takada H , Sasagawa Y , Yoshimura M , et al. Single‐cell transcriptomics uncovers EGFR signaling‐mediated gastric progenitor cell differentiation in stomach homeostasis. Nat Commun. 2023;14(1):3750. 37386010 10.1038/s41467-023-39113-0 PMC10310803

[44] Dijkstra KK , Cattaneo CM , Weeber F , et al. Generation of tumor‐reactive T cells by co‐culture of peripheral blood lymphocytes and tumor organoids. Cell. 2018;174(6):1586‐1598. e12. 30100188 10.1016/j.cell.2018.07.009 PMC6558289

[45] Cattaneo CM , Dijkstra KK , Fanchi LF , et al. Tumor organoid‐T‐cell coculture systems. Nat Protoc. 2020;15(1):15‐39. 31853056 10.1038/s41596-019-0232-9 PMC7610702

[46] Neal JT , Li X , Zhu J , et al. Organoid modeling of the tumor immune microenvironment. Cell. 2018;175(7):1972‐1988. e16. 30550791 10.1016/j.cell.2018.11.021 PMC6656687

[47] Holloway EM , Wu JH , Czerwinski M , et al. Differentiation of human intestinal organoids with endogenous vascular endothelial cells. Dev Cell. 2020;54(4):516‐528. e7. 32841595 10.1016/j.devcel.2020.07.023 PMC7480827

[48] Palikuqi B , Nguyen DT , Li G , et al. Adaptable haemodynamic endothelial cells for organogenesis and tumorigenesis. Nature. 2020;585(7825):426‐432. 32908310 10.1038/s41586-020-2712-z PMC7480005

[49] Lei C , Sun R , Xu G , et al. Enteric VIP‐producing neurons maintain gut microbiota homeostasis through regulating epithelium fucosylation. Cell Host Microbe. 2022;30(10):1417‐1434. e8. 36150396 10.1016/j.chom.2022.09.001 PMC9588764

[50] Cao YG , Bae S , Villarreal J , et al. Faecalibaculum rodentium remodels retinoic acid signaling to govern eosinophil‐dependent intestinal epithelial homeostasis. Cell Host Microbe. 2022;30(9):1295‐1310. e8. 35985335 10.1016/j.chom.2022.07.015 PMC9481734

[51] Haber AL , Biton M , Rogel N , et al. A single‐cell survey of the small intestinal epithelium. Nature. 2017;551(7680):333‐339. 29144463 10.1038/nature24489 PMC6022292

[52] Galeano Nino JL, Wu H , LaCourse KD , et al. Effect of the intratumoral microbiota on spatial and cellular heterogeneity in cancer. Nature. 2022;611(7937):810‐817. 36385528 10.1038/s41586-022-05435-0 PMC9684076

[53] Sun L , Rollins D , Qi Y , et al. TNFalpha regulates intestinal organoids from mice with both defined and conventional microbiota. Int J Biol Macromol. 2020;164:548‐556. 32693143 10.1016/j.ijbiomac.2020.07.176 PMC7657954

[54] Reding B , Carter P , Qi Y , et al. Manipulate intestinal organoids with niobium carbide nanosheets. J Biomed Mater Res A. 2021;109(4):479‐487. 32506610 10.1002/jbm.a.37032

[55] Tong T , Qi Y , Bussiere LD , et al. Transport of artificial virus‐like nanocarriers through intestinal monolayers via microfold cells. Nanoscale. 2020;12(30):16339‐16347. 32725029 10.1039/d0nr03680c

[56] Qi Y , Shi E , Peroutka‐Bigus N , et al. Ex vivo study of telluride nanowires in Minigut. J Biomed Nanotechnol. 2018;14(5):978‐986. 29883567 10.1166/jbn.2018.2578

[57] Fair KL , Colquhoun J , Hannan NRF . Intestinal organoids for modelling intestinal development and disease. Philos Trans R Soc Lond B Biol Sci. 2018;373(1750):20170217. 29786552 10.1098/rstb.2017.0217 PMC5974440

[58] Hansson GC . Mucins and the microbiome. Annu Rev Biochem. 2020;89:769‐793. 32243763 10.1146/annurev-biochem-011520-105053 PMC8442341

[59] Paone P , Cani PD . Mucus barrier, mucins and gut microbiota: the expected slimy partners? Gut. 2020;69(12):2232‐2243. 32917747 10.1136/gutjnl-2020-322260 PMC7677487

[60] Gonyar LA , Smith RM , Giron JA , Zachos NC , Ruiz‐Perez F , Nataro JP . Aggregative adherence fimbriae II of enteroaggregative escherichia coli are required for adherence and barrier disruption during infection of human colonoids. Infect Immun. 2020;88(9):e00176. 32631917 10.1128/IAI.00176-20 PMC7440768

[61] Sontheimer‐Phelps A , Chou DB , Tovaglieri A , et al. Human colon‐on‐a‐chip enables continuous in vitro analysis of colon mucus layer accumulation and physiology. Cell Mol Gastroenterol Hepatol. 2020;9(3):507‐526. 31778828 10.1016/j.jcmgh.2019.11.008 PMC7036549

[62] Hill DR , Huang S , Nagy MS , et al. Bacterial colonization stimulates a complex physiological response in the immature human intestinal epithelium. eLife. 2017;6:e29132. 29110754 10.7554/eLife.29132 PMC5711377

[63] Min S , Than N , Shin YC , et al. Live probiotic bacteria administered in a pathomimetic Leaky Gut Chip ameliorate impaired epithelial barrier and mucosal inflammation. Sci Rep. 2022;12(1):22641. 36587177 10.1038/s41598-022-27300-w PMC9805460

[64] Son YS , Ki SJ , Thanavel R , et al. Maturation of human intestinal organoids in vitro facilitates colonization by commensal lactobacilli by reinforcing the mucus layer. FASEB J. 2020;34(8):9899‐9910. 32602623 10.1096/fj.202000063R

[65] Wlodarska M , Luo C , Kolde R , et al. Indoleacrylic acid produced by commensal peptostreptococcus species suppresses inflammation. Cell Host Microbe. 2017;22(1):25‐37. e6. 28704649 10.1016/j.chom.2017.06.007 PMC5672633

[66] Clevers H . The intestinal crypt, a prototype stem cell compartment. Cell. 2013;154(2):274‐284. 23870119 10.1016/j.cell.2013.07.004

[67] Sato T , Clevers H . Growing self‐organizing mini‐guts from a single intestinal stem cell: mechanism and applications. Science. 2013;340(6137):1190‐1194. 23744940 10.1126/science.1234852

[68] Leushacke M , Barker N . Ex vivo culture of the intestinal epithelium: strategies and applications. Gut. 2014;63(8):1345‐1354. 24841573 10.1136/gutjnl-2014-307204

[69] Wilson SS , Tocchi A , Holly MK , Parks WC , Smith JG . A small intestinal organoid model of non‐invasive enteric pathogen‐epithelial cell interactions. Mucosal Immunol. 2015;8(2):352‐361. 25118165 10.1038/mi.2014.72 PMC4326599

[70] Clark HR , McKenney C , Livingston NM , et al. Epigenetically regulated digital signaling defines epithelial innate immunity at the tissue level. Nat Commun. 2021;12(1):1836. 33758175 10.1038/s41467-021-22070-x PMC7988009

[71] Clasen SJ , Bell MEW , Borbon A , et al. Silent recognition of flagellins from human gut commensal bacteria by Toll‐like receptor 5. Sci Immunol. 2023;8(79):eabq7001. 36608151 10.1126/sciimmunol.abq7001

[72] Wang Y , Chiang IL , Ohara TE , et al. Long‐term culture captures injury‐repair cycles of colonic stem cells. Cell. 2019;179(5):1144‐1159. e15. 31708126 10.1016/j.cell.2019.10.015 PMC6904908

[73] Ranganathan S , Smith EM , Foulke‐Abel JD , Barry EM . Research in a time of enteroids and organoids: how the human gut model has transformed the study of enteric bacterial pathogens. Gut Microbes. 2020;12(1):1795492. 10.1080/19490976.2020.1795389 PMC7524385 32795243

[74] Gazzaniga FS , Camacho DM , Wu M , et al. Harnessing colon chip technology to identify commensal bacteria that promote host tolerance to infection. Front Cell Infect Microbiol. 2021;11:638014. 33777849 10.3389/fcimb.2021.638014 PMC7996096

[75] Fasciano AC , Dasanayake GS , Estes MK , et al. Yersinia pseudotuberculosis YopE prevents uptake by M cells and instigates M cell extrusion in human ileal enteroid‐derived monolayers. Gut Microbes. 2021;13(1):1988390. 34793276 10.1080/19490976.2021.1988390 PMC8604394

[76] Zhou C , Zhang Y , Bassey A , Huang J , Zou Y , Ye K . Expansion of intestinal secretory cell population induced by listeria monocytogenes infection: accompanied with the inhibition of NOTCH pathway. Front Cell Infect Microbiol. 2022;12:793335. 35402308 10.3389/fcimb.2022.793335 PMC8990097

[77] Huang J , Zhou C , Zhou G , Li H , Ye K . Effect of Listeria monocytogenes on intestinal stem cells in the co‐culture model of small intestinal organoids. Microb Pathog. 2021;153:104776. 33548482 10.1016/j.micpath.2021.104776

[78] Mileto SJ , Jarde T , Childress KO , et al. Clostridioides difficile infection damages colonic stem cells via TcdB, impairing epithelial repair and recovery from disease. Proc Natl Acad Sci USA. 2020;117(14):8064‐8073. 32198200 10.1073/pnas.1915255117 PMC7149309

[79] Engevik MA , Danhof HA , Chang‐Graham AL , et al. Human intestinal enteroids as a model of Clostridioides difficile‐induced enteritis. Am J Physiol Gastrointest Liver Physiol. 2020;318(5):G870‐G888. 32223302 10.1152/ajpgi.00045.2020 PMC7272722

[80] Matson V , Chervin CS , Gajewski TF . Cancer and the microbiome‐influence of the commensal microbiota on cancer, immune responses, and immunotherapy. Gastroenterology. 2021;160(2):600‐613. 33253684 10.1053/j.gastro.2020.11.041 PMC8409239

[81] Levy M , Kolodziejczyk AA , Thaiss CA , Elinav E . Dysbiosis and the immune system. Nat Rev Immunol. 2017;17(4):219‐232. 28260787 10.1038/nri.2017.7

[82] Fang H , Huang Y , Luo Y , et al. SIRT1 induces the accumulation of TAMs at colorectal cancer tumor sites via the CXCR4/CXCL12 axis. Cell Immunol. 2022;371:104458. 34847407 10.1016/j.cellimm.2021.104458

[83] Hentschel V , Seufferlein T , Armacki M . Intestinal organoids in coculture: redefining the boundaries of gut mucosa ex vivo modeling. Am J Physiol Gastrointest Liver Physiol. 2021;321(6):G693‐G704. 34643092 10.1152/ajpgi.00043.2021

[84] Engevik MA , Ruan W , Esparza M , et al. Immunomodulation of dendritic cells by Lactobacillus reuteri surface components and metabolites. Physiol Rep. 2021;9(2):e14719. 33463911 10.14814/phy2.14719 PMC7814497

[85] Noel G , Baetz NW , Staab JF , et al. A primary human macrophage‐enteroid co‐culture model to investigate mucosal gut physiology and host‐pathogen interactions. Sci Rep. 2017;7:45270. 28345602 10.1038/srep45270 PMC5366908

[86] Gao Y , Bi D , Xie R , et al. Fusobacterium nucleatum enhances the efficacy of PD‐L1 blockade in colorectal cancer. Signal Transduct Target Ther. 2021;6(1):398. 34795206 10.1038/s41392-021-00795-x PMC8602417

[87] Lindemans CA , Calafiore M , Mertelsmann AM , et al. Interleukin‐22 promotes intestinal‐stem‐cell‐mediated epithelial regeneration. Nature. 2015;528(7583):560‐564. 26649819 10.1038/nature16460 PMC4720437

[88] Fu Z , Dean JW , Xiong L , et al. Mitochondrial transcription factor A in RORgammat(+) lymphocytes regulate small intestine homeostasis and metabolism. Nat Commun. 2021;12(1):4462. 34294718 10.1038/s41467-021-24755-9 PMC8298438

[89] Hou Q , Ye L , Liu H , et al. Correction: lactobacillus accelerates ISCs regeneration to protect the integrity of intestinal mucosa through activation of STAT3 signaling pathway induced by LPLs secretion of IL‐22. Cell Death Differ. 2021;28(6):2025‐2027. 33087876 10.1038/s41418-020-00630-w PMC8184977

[90] Zhu P , Lu T , Wu J , et al. Gut microbiota drives macrophage‐dependent self‐renewal of intestinal stem cells via niche enteric serotonergic neurons. Cell Res. 2022;32(6):555‐569. 35379903 10.1038/s41422-022-00645-7 PMC9160288

[91] Murota Y , Jobin C . Bacteria break barrier to promote metastasis. Cancer Cell. 2021;39(5):598‐600. 33891891 10.1016/j.ccell.2021.03.009 PMC8982444

[92] Koike H , Iwasawa K , Ouchi R , et al. Modelling human hepato‐biliary‐pancreatic organogenesis from the foregut‐midgut boundary. Nature. 2019;574(7776):112‐116. 31554966 10.1038/s41586-019-1598-0 PMC7643931

[93] Marsee A , Roos FJM , Verstegen MMA , et al. Building consensus on definition and nomenclature of hepatic, pancreatic, and biliary organoids. Cell Stem Cell. 2021;28(5):816‐832. 33961769 10.1016/j.stem.2021.04.005 PMC11699540

[94] Meir M , Kannapin F , Diefenbacher M , et al. Intestinal epithelial barrier maturation by enteric glial cells is GDNF‐dependent. Int J Mol Sci. 2021;22(4):1887. 33672854 10.3390/ijms22041887 PMC7917776

[95] Nakamoto N , Sasaki N , Aoki R , et al. Gut pathobionts underlie intestinal barrier dysfunction and liver T helper 17 cell immune response in primary sclerosing cholangitis. Nat Microbiol. 2019;4(3):492‐503. 30643240 10.1038/s41564-018-0333-1

[96] Hassan M , Moghadamrad S , Sorribas M , et al. Paneth cells promote angiogenesis and regulate portal hypertension in response to microbial signals. J Hepatol. 2020;73(3):628‐639. 32205193 10.1016/j.jhep.2020.03.019

[97] Lee KS , Lee J , Lee P , et al. Inhibition of O‐GlcNAcylation protects from Shiga toxin‐mediated cell injury and lethality in host. EMBO Mol Med. 2022;14(1):e14678. 34842355 10.15252/emmm.202114678 PMC8749473

[98] Xiong X , Tian S , Yang P , et al. Emerging enterococcus pore‐forming toxins with MHC/HLA‐I as receptors. Cell. 2022;185(7):1157‐1171. e22. 35259335 10.1016/j.cell.2022.02.002 PMC8978092

[99] Fazio A , Rosenstiel P . Language of a long‐term relationship: bacterial inositols and the intestinal epithelium. Cell Metab. 2020;32(4):509‐511. 33027672 10.1016/j.cmet.2020.09.015

[100] Wu SE , Hashimoto‐Hill S , Woo V , et al. Microbiota‐derived metabolite promotes HDAC3 activity in the gut. Nature. 2020;586(7827):108‐112. 32731255 10.1038/s41586-020-2604-2 PMC7529926

[101] Lukovac S , Belzer C , Pellis L , et al. Differential modulation by Akkermansia muciniphila and Faecalibacterium prausnitzii of host peripheral lipid metabolism and histone acetylation in mouse gut organoids. mBio. 2014;5(4):e01438. 25118238 10.1128/mBio.01438-14 PMC4145684

[102] Fawad JA , Luzader DH , Hanson GF , et al. Histone deacetylase inhibition by gut microbe‐generated short‐chain fatty acids entrains intestinal epithelial circadian rhythms. Gastroenterology. 2022;163(5):1377‐1390. e11. 35934064 10.1053/j.gastro.2022.07.051 PMC11551968

[103] Hinrichsen F , Hamm J , Westermann M , et al. Microbial regulation of hexokinase 2 links mitochondrial metabolism and cell death in colitis. Cell Metab. 2021;33(12):2355‐2366. e8. 34847376 10.1016/j.cmet.2021.11.004

[104] Abo H , Chassaing B , Harusato A , et al. Erythroid differentiation regulator‐1 induced by microbiota in early life drives intestinal stem cell proliferation and regeneration. Nat Commun. 2020;11(1):513. 31980634 10.1038/s41467-019-14258-z PMC6981263

[105] Park M , Kwon J , Shin HJ , et al. Butyrate enhances the efficacy of radiotherapy via FOXO3A in colorectal cancer patientderived organoids. Int J Oncol. 2020;57(6):1307‐1318. 33173975 10.3892/ijo.2020.5132 PMC7646587

[106] Liu TC , Kern JT , Jain U , et al. Western diet induces Paneth cell defects through microbiome alterations and farnesoid X receptor and type I interferon activation. Cell Host Microbe. 2021;29(6):988‐1001. e6. 34010595 10.1016/j.chom.2021.04.004 PMC8192497

[107] Iftekhar A , Berger H , Bouznad N , et al. Genomic aberrations after short‐term exposure to colibactin‐producing E. coli transform primary colon epithelial cells. Nat Commun. 2021;12(1):1003. 33579932 10.1038/s41467-021-21162-y PMC7881031

[108] Pleguezuelos‐Manzano C , Puschhof J , Rosendahl Huber A , et al. Mutational signature in colorectal cancer caused by genotoxic pks(+) E. coli. Nature. 2020;580(7802):269‐273. 32106218 10.1038/s41586-020-2080-8 PMC8142898

[109] Kadosh E , Snir‐Alkalay I , Venkatachalam A , et al. The gut microbiome switches mutant p53 from tumour‐suppressive to oncogenic. Nature. 2020;586(7827):133‐138. 32728212 10.1038/s41586-020-2541-0 PMC7116712

[110] Giri R , Hoedt EC , Khushi S , et al. Secreted NF‐kappaB suppressive microbial metabolites modulate gut inflammation. Cell Rep. 2022;39(2):110646. 35417687 10.1016/j.celrep.2022.110646

[111] Weersma RK , Zhernakova A , Fu J . Interaction between drugs and the gut microbiome. Gut. 2020;69(8):1510‐1519. 32409589 10.1136/gutjnl-2019-320204 PMC7398478

[112] Pasolli E , Asnicar F , Manara S , et al. Extensive unexplored human microbiome diversity revealed by over 150,000 genomes from metagenomes spanning age, geography, and lifestyle. Cell. 2019;176(3):649‐662. e20. 30661755 10.1016/j.cell.2019.01.001 PMC6349461

[113] Brito IL , Gurry T , Zhao S , et al. Transmission of human‐associated microbiota along family and social networks. Nat Microbiol. 2019;4(6):964‐971. 30911128 10.1038/s41564-019-0409-6 PMC7450247

[114] Zmora N , Suez J , Elinav E . You are what you eat: diet, health and the gut microbiota. Nat Rev Gastroenterol Hepatol. 2019;16(1):35‐56. 30262901 10.1038/s41575-018-0061-2

[115] Jorgensen I , Rayamajhi M , Miao EA . Programmed cell death as a defence against infection. Nat Rev Immunol. 2017;17(3):151‐164. 28138137 10.1038/nri.2016.147 PMC5328506

[116] Anderson CJ , Medina CB , Barron BJ , et al. Microbes exploit death‐induced nutrient release by gut epithelial cells. Nature. 2021;596(7871):262‐267. 34349263 10.1038/s41586-021-03785-9

[117] Kurilshikov A , Medina‐Gomez C , Bacigalupe R , et al. Large‐scale association analyses identify host factors influencing human gut microbiome composition. Nat Genet. 2021;53(2):156‐165. 33462485 10.1038/s41588-020-00763-1 PMC8515199

[118] Jamwal DR , Laubitz D , Harrison CA , et al. Intestinal epithelial expression of MHCII determines severity of chemical, T‐cell‐induced, and infectious colitis in mice. Gastroenterology. 2020;159(4):1342‐1356. e6. 32589883 10.1053/j.gastro.2020.06.049 PMC9190026

[119] Sharma A , Achi SC , Ibeawuchi SR , et al. The crosstalk between microbial sensors ELMO1 and NOD2 shape intestinal immune responses. Virulence. 2023;14(1):2171690. 36694274 10.1080/21505594.2023.2171690 PMC9980453

[120] Sugimura N , Li Q , Chu ESH , et al. Lactobacillus gallinarum modulates the gut microbiota and produces anti‐cancer metabolites to protect against colorectal tumourigenesis. Gut. 2021;71(10):2011‐2021. 34937766 10.1136/gutjnl-2020-323951 PMC9484392

[121] Banerjee A , Herring CA , Chen B , et al. Succinate produced by intestinal microbes promotes specification of tuft cells to suppress ileal inflammation. Gastroenterology. 2020;159(6):2101‐2115. e5. 32828819 10.1053/j.gastro.2020.08.029 PMC7725941

[122] Das NK , Schwartz AJ , Barthel G , et al. Microbial metabolite signaling is required for systemic iron homeostasis. Cell Metab. 2020;31(1):115‐130. e6. 31708445 10.1016/j.cmet.2019.10.005 PMC6949377

[123] Engevik MA , Aihara E , Montrose MH , Shull GE , Hassett DJ , Worrell RT . Loss of NHE3 alters gut microbiota composition and influences Bacteroides thetaiotaomicron growth. Am J Physiol Gastrointest Liver Physiol. 2013;305(10):G697‐G711. 24072680 10.1152/ajpgi.00184.2013 PMC3840232

[124] Sasaki N , Miyamoto K , Maslowski KM , Ohno H , Kanai T , Sato T . Development of a scalable coculture system for gut anaerobes and human colon epithelium. Gastroenterology. 2020;159(1):388‐390. e5. 32199883 10.1053/j.gastro.2020.03.021

[125] Al Shihabi A, Davarifar A , Nguyen HTL , et al. Personalized chordoma organoids for drug discovery studies. Sci Adv. 2022;8(7):eabl3674. 35171675 10.1126/sciadv.abl3674 PMC8849332

[126] Tiriac H , Bucobo JC , Tzimas D , et al. Successful creation of pancreatic cancer organoids by means of EUS‐guided fine‐needle biopsy sampling for personalized cancer treatment. Gastrointest Endosc. 2018;87(6):1474‐1480. 29325707 10.1016/j.gie.2017.12.032 PMC6143289

[127] Kopper O , de Witte CJ , Lohmussaar K , et al. An organoid platform for ovarian cancer captures intra‐ and interpatient heterogeneity. Nat Med. 2019;25(5):838‐849. 31011202 10.1038/s41591-019-0422-6

[128] Seino T , Kawasaki S , Shimokawa M , et al. Human pancreatic tumor organoids reveal loss of stem cell niche factor dependence during disease progression. Cell Stem Cell. 2018;22(3):454‐467. e6. 29337182 10.1016/j.stem.2017.12.009

[129] Zhao Z , Chen X , Dowbaj AM , et al. Organoids. Nat Rev Methods Primers. 2022;2:94. 37325195 10.1038/s43586-022-00174-y PMC10270325

[130] Gobert AP , Smith TM , Latour YL , et al. Hypusination maintains intestinal homeostasis and prevents colitis and carcinogenesis by enhancing aldehyde detoxification. Gastroenterology. 2023;165(3):656‐669. e8. 37271289 10.1053/j.gastro.2023.05.041 PMC10527201

[131] Vande Voorde J , Steven RT , Najumudeen AK , et al. Metabolic profiling stratifies colorectal cancer and reveals adenosylhomocysteinase as a therapeutic target. Nat Metab. 2023;5(8):1303‐1318. 37580540 10.1038/s42255-023-00857-0 PMC10447251

[132] An Y , Wang C , Fan B , et al. LSR targets YAP to modulate intestinal Paneth cell differentiation. Cell Rep. 2023;42(9):113118. 37703178 10.1016/j.celrep.2023.113118

[133] Chang L , Jung NY , Atari A , et al. Systematic profiling of conditional pathway activation identifies context‐dependent synthetic lethalities. Nat Genet. 2023;55(10):1709‐1720. 37749246 10.1038/s41588-023-01515-7

[134] Strating E , Verhagen MP , Wensink E , et al. Co‐cultures of colon cancer cells and cancer‐associated fibroblasts recapitulate the aggressive features of mesenchymal‐like colon cancer. Front Immunol. 2023;14:1053920. 37261365 10.3389/fimmu.2023.1053920 PMC10228738

[135] Li N , Zhu Q , Tian Y , et al. Mapping and modeling human colorectal carcinoma interactions with the tumor microenvironment. Nat Commun. 2023;14(1):7915. 38036590 10.1038/s41467-023-43746-6 PMC10689473

[136] Jiang Y , Zhao H , Kong S , et al. Establishing mouse and human oral esophageal organoids to investigate the tumor immune response. Dis Model Mech. 2024;17(1):dmm050319. 38258518 10.1242/dmm.050319 PMC10846528

[137] Ko KP , Huang Y , Zhang S , et al. Key genetic determinants driving esophageal squamous cell carcinoma initiation and immune evasion. Gastroenterology. 2023;165(3):613‐628. e20. 37257519 10.1053/j.gastro.2023.05.030 PMC10527250

[138] Baumdick ME , Niehrs A , Degenhardt F , et al. HLA‐DP on epithelial cells enables tissue damage by NKp44(+) natural killer cells in ulcerative colitis. Gastroenterology. 2023;165(4):946‐962. e13. 37454979 10.1053/j.gastro.2023.06.034 PMC10529779

[139] Farin HF , Mosa MH , Ndreshkjana B , et al. Colorectal cancer organoid‐stroma biobank allows subtype‐specific assessment of individualized therapy responses. Cancer Discov. 2023;13(10):2192‐2211. 37489084 10.1158/2159-8290.CD-23-0050 PMC10551667

[140] Kawaguchi T , Okamoto K , Fujimoto S , et al. Lansoprazole inhibits the development of sessile serrated lesions by inducing G1 arrest via Skp2/p27 signaling pathway. J Gastroenterol. 2024;59(1):11‐23. 37989907 10.1007/s00535-023-02052-0

[141] Takeuchi C , Yamashita S , Liu YY , et al. Precancerous nature of intestinal metaplasia with increased chance of conversion and accelerated DNA methylation. Gut. 2024;73(2):255‐267. 37751933 10.1136/gutjnl-2023-329492

[142] Karlsson K , Przybilla MJ , Kotler E , et al. Deterministic evolution and stringent selection during preneoplasia. Nature. 2023;618(7964):383‐393. 37258665 10.1038/s41586-023-06102-8 PMC10247377

[143] Wilson MR , Jiang Y , Villalta PW , et al. The human gut bacterial genotoxin colibactin alkylates DNA. Science. 2019;363(6428):eaar7785. 30765538 10.1126/science.aar7785 PMC6407708

[144] Yu LC , Wei SC , Li YH , et al. Invasive pathobionts contribute to colon cancer initiation by counterbalancing epithelial antimicrobial responses. Cell Mol Gastroenterol Hepatol. 2022;13(1):57‐79. 34418587 10.1016/j.jcmgh.2021.08.007 PMC8600093

[145] Wroblewski LE , Peek RM Jr . Helicobacter pylori and gastric cancer: factors that modulate disease risk. Clin Microbiol Rev. 2010;23(4):713‐739. 20930071 10.1128/CMR.00011-10 PMC2952980

[146] He J , Nascakova Z , Leary P , et al. Inactivation of the tumor suppressor gene Apc synergizes with H. pylori to induce DNA damage in murine gastric stem and progenitor cells. Sci Adv. 2023;9(46):eadh0322. 37967175 10.1126/sciadv.adh0322 PMC10651120

[147] Kim TY , Kim S , Kim Y , et al. A high‐fat diet activates the BAs‐FXR axis and triggers cancer‐associated fibroblast properties in the colon. Cell Mol Gastroenterol Hepatol. 2022;13(4):1141‐1159. 34971821 10.1016/j.jcmgh.2021.12.015 PMC8873938

[148] Engevik MA , Danhof HA , Ruan W , et al. Fusobacterium nucleatum secretes outer membrane vesicles and promotes intestinal inflammation. mBio. 2021;12(2):e02706. 33653893 10.1128/mBio.02706-20 PMC8092269

[149] Ding W , Marx OM , Mankarious MM , Koltun WA , Yochum GS . Disease severity impairs generation of intestinal organoid cultures from inflammatory bowel disease patients. J Surg Res. 2024;293:187‐195. 37776721 10.1016/j.jss.2023.08.027

[150] Arnauts K , Sudhakar P , Verstockt S , et al. Microbiota, not host origin drives ex vivo intestinal epithelial responses. Gut Microbes. 2022;14(1):2089003. 35758256 10.1080/19490976.2022.2089003 PMC9235885

[151] Iribarren C , Nordlander S , Sundin J , et al. Fecal luminal factors from patients with irritable bowel syndrome induce distinct gene expression of colonoids. Neurogastroenterol Motil. 2022;34(10):e14390. 35485994 10.1111/nmo.14390 PMC9786662

[152] Gao J , Xiong T , Grabauskas G , Owyang C . Mucosal serotonin reuptake transporter expression in irritable bowel syndrome is modulated by gut microbiota via mast cell‐prostaglandin E2. Gastroenterology. 2022;162(7):1962‐1974. e6. 35167867 10.1053/j.gastro.2022.02.016 PMC9117493

[153] Grabauskas G , Gao J , Wu X , Zhou SY , Turgeon DK , Owyang C . Gut microbiota alter visceral pain sensation and inflammation via modulation of synthesis of resolvin D1 in colonic tuft cells. Gastroenterology. 2022. 10.1053/j.gastro.2022.07.053 PMC9898459 35934059

[154] Hosmillo M , Chaudhry Y , Nayak K , et al. Norovirus replication in human intestinal epithelial cells is restricted by the interferon‐induced JAK/STAT signaling pathway and RNA polymerase II‐mediated transcriptional responses. mBio. 2020;11(2):e00215. 32184238 10.1128/mBio.00215-20 PMC7078467

[155] Haga K , Ettayebi K , Tenge VR , et al. Genetic manipulation of human intestinal enteroids demonstrates the necessity of a functional fucosyltransferase 2 gene for secretor‐dependent human norovirus infection. mBio. 2020;11(2):e00251. 32184242 10.1128/mBio.00251-20 PMC7078471

[156] Lamers MM , Beumer J , van der Vaart J , et al. SARS‐CoV‐2 productively infects human gut enterocytes. Science. 2020;369(6499):50‐54. 32358202 10.1126/science.abc1669 PMC7199907

[157] Han Y , Duan X , Yang L , et al. Identification of SARS‐CoV‐2 inhibitors using lung and colonic organoids. Nature. 2021;589(7841):270‐275. 33116299 10.1038/s41586-020-2901-9 PMC8034380

[158] Tate JE , Burton AH , Boschi‐Pinto C , et al. 2008 estimate of worldwide rotavirus‐associated mortality in children younger than 5 years before the introduction of universal rotavirus vaccination programmes: a systematic review and meta‐analysis. Lancet Infect Dis. 2012;12(2):136‐141. 22030330 10.1016/S1473-3099(11)70253-5

[159] Saxena K , Blutt SE , Ettayebi K , et al. Human intestinal enteroids: a new model to study human rotavirus infection, host restriction, and pathophysiology. J Virol. 2016;90(1):43‐56. 26446608 10.1128/JVI.01930-15 PMC4702582

[160] Wilke G , Funkhouser‐Jones LJ , Wang Y , et al. A stem‐cell‐derived platform enables complete cryptosporidium development in vitro and genetic tractability. Cell Host Microbe. 2019;26(1):123‐134. e8. 31231046 10.1016/j.chom.2019.05.007 PMC6617391

[161] Griger J , Widholz SA , Jesinghaus M , et al. An integrated cellular and molecular model of gastric neuroendocrine cancer evolution highlights therapeutic targets. Cancer Cell. 2023;41(7):1327‐1344. e10. 37352862 10.1016/j.ccell.2023.06.001

[162] Kawasaki K , Toshimitsu K , Matano M , et al. An organoid biobank of neuroendocrine neoplasms enables genotype‐phenotype mapping. Cell. 2020;183(5):1420‐1435. e21. 33159857 10.1016/j.cell.2020.10.023

[163] Giri AK , Aavikko M , Wartiovaara L , et al. Genome‐Wide association study identifies 4 novel risk loci for small intestinal neuroendocrine tumors including a missense mutation in LGR5. Gastroenterology. 2023;165(4):861‐873. 37453564 10.1053/j.gastro.2023.06.031

[164] Poplaski V , Bomidi C , Kambal A , et al. Human intestinal organoids from Cronkhite‐Canada syndrome patients reveal link between serotonin and proliferation. J Clin Invest. 2023;133(21):e166884. 37909332 10.1172/JCI166884 PMC10617781

[165] Deng F , Zhao BC , Yang X , et al. The gut microbiota metabolite capsiate promotes Gpx4 expression by activating TRPV1 to inhibit intestinal ischemia reperfusion‐induced ferroptosis. Gut Microbes. 2021;13(1):1‐21. 10.1080/19490976.2021.1902719 PMC8009132 33779497

[166] Rosselot AE , Park M , Kim M , et al. Ontogeny and function of the circadian clock in intestinal organoids. EMBO J. 2022;41(2):e106973. 34704277 10.15252/embj.2020106973 PMC8762567

[167] Frazier K , Kambal A , Zale EA , et al. High‐fat diet disrupts REG3gamma and gut microbial rhythms promoting metabolic dysfunction. Cell Host Microbe. 2022;30(6):809‐823. e6. 35439436 10.1016/j.chom.2022.03.030 PMC9281554

[168] Zhou C , Fang X , Xu J , et al. Bifidobacterium longum alleviates irritable bowel syndrome‐related visceral hypersensitivity and microbiota dysbiosis via Paneth cell regulation. Gut Microbes. 2020;12(1):1782156. 32584650 10.1080/19490976.2020.1782156 PMC7524277

[169] Kim S , Shin YC , Kim TY , et al. Mucin degrader Akkermansia muciniphila accelerates intestinal stem cell‐mediated epithelial development. Gut Microbes. 2021;13(1):1‐20. 10.1080/19490976.2021.1892441 PMC7946046 33678130

[170] Conte M , Nigro F , Porpora M , et al. Gliadin peptide P31‐43 induces mTOR/NFkbeta activation and reduces autophagy: the role of Lactobacillus paracasei CBA L74 Postbiotc. Int J Mol Sci. 2022;23(7):3655. 35409015 10.3390/ijms23073655 PMC8999065

[171] Deng F , Hu JJ , Yang X , et al. Gut microbial metabolite pravastatin attenuates intestinal ischemia/reperfusion injury through promoting IL‐13 release from type II innate lymphoid cells via IL‐33/ST2 signaling. Front Immunol. 2021;12:704836. 34650552 10.3389/fimmu.2021.704836 PMC8505964

[172] Sittipo P , Pham HQ , Park CE , et al. Irradiation‐Induced intestinal damage is recovered by the indigenous gut bacteria Lactobacillus acidophilus. Front Cell Infect Microbiol. 2020;10:415. 32974214 10.3389/fcimb.2020.00415 PMC7461978

[173] Wu H , Xie S , Miao J , et al. Lactobacillus reuteri maintains intestinal epithelial regeneration and repairs damaged intestinal mucosa. Gut Microbes. 2020;11(4):997‐1014. 32138622 10.1080/19490976.2020.1734423 PMC7524370

[174] Darby TM , Naudin CR , Luo L , Jones RM . Lactobacillus rhamnosus GG‐induced expression of leptin in the intestine orchestrates epithelial cell proliferation. Cell Mol Gastroenterol Hepatol. 2020;9(4):627‐639. 31874255 10.1016/j.jcmgh.2019.12.004 PMC7160578

[175] Sorrentino G , Perino A , Yildiz E , et al. Bile acids signal via TGR5 to activate intestinal stem cells and epithelial regeneration. Gastroenterology. 2020;159(3):956‐968. e8. 32485177 10.1053/j.gastro.2020.05.067

[176] Bajic D , Niemann A , Hillmer AK , et al. Gut microbiota‐derived propionate regulates the expression of Reg3 mucosal lectins and ameliorates experimental colitis in mice. J Crohns Colitis. 2020;14(10):1462‐1472. 32227170 10.1093/ecco-jcc/jjaa065 PMC8921751

[177] Lee YS , Kim TY , Kim Y , et al. Microbiota‐derived lactate accelerates intestinal stem‐cell‐mediated epithelial development. Cell Host Microbe. 2018;24(6):833‐846. e6. 30543778 10.1016/j.chom.2018.11.002

[178] Phan N , Hong JJ , Tofig B , et al. A simple high‐throughput approach identifies actionable drug sensitivities in patient‐derived tumor organoids. Commun Biol. 2019;2:78. 30820473 10.1038/s42003-019-0305-x PMC6389967

[179] Tran TQ , Hanse EA , Habowski AN , et al. alpha‐Ketoglutarate attenuates Wnt signaling and drives differentiation in colorectal cancer. Nat Cancer. 2020;1(3):345‐358. 32832918 10.1038/s43018-020-0035-5 PMC7442208

[180] Hamed SA , Mohan A , Navaneetha Krishnan S , et al. Butyrate reduces adherent‐invasive E. coli‐evoked disruption of epithelial mitochondrial morphology and barrier function: involvement of free fatty acid receptor 3. Gut Microbes. 2023;15(2):2281011. 38078655 10.1080/19490976.2023.2281011 PMC10730202

[181] Gou H , Su H , Liu D , et al. Traditional medicine pien tze huang suppresses colorectal tumorigenesis through restoring gut microbiota and metabolites. Gastroenterology. 2023;165(6):1404‐1419. 37704113 10.1053/j.gastro.2023.08.052

[182] Frazer LC , Yamaguchi Y , Jania CM , et al. Microfluidic model of necrotizing enterocolitis incorporating human neonatal intestinal enteroids and a dysbiotic microbiome. J Vis Exp. 2023;(197). 10.3791/65605 PMC11003451 37590536

[183] Terzo E , Apte SA , Padhye S , et al. A novel class of ribosome modulating agents exploits cancer ribosome heterogeneity to selectively target the CMS2 subtype of colorectal cancer. Cancer Res Commun. 2023;3(6):969‐979. 37377612 10.1158/2767-9764.CRC-22-0469 PMC10241187

[184] Cruz‐Acuna R , Kariuki SW , Sugiura K , et al. Engineered hydrogel reveals contribution of matrix mechanics to esophageal adenocarcinoma and identifies matrix‐activated therapeutic targets. J Clin Invest. 2023;133(23):e168146. 37788109 10.1172/JCI168146 PMC10688988

[185] Hu S , Xia K , Huang X , et al. Multifunctional CaCO(3)@Cur@QTX125@HA nanoparticles for effectively inhibiting growth of colorectal cancer cells. J Nanobiotechnology. 2023;21(1):353. 37773145 10.1186/s12951-023-02104-w PMC10543835

[186] Schmache T , Fohgrub J , Klimova A , et al. Stratifying esophago‐gastric cancer treatment using a patient‐derived organoid‐based threshold. Mol Cancer. 2024;23(1):10. 38200602 10.1186/s12943-023-01919-3 PMC10777586

[187] Hasselluhn MC , Schlosser D , Versemann L , et al. An NFATc1/SMAD3/cJUN complex restricted to SMAD4‐deficient pancreatic cancer guides rational therapies. Gastroenterology. 2024;166(2):298‐312. e14.37913894 10.1053/j.gastro.2023.10.026

[188] Wang D , Nakayama M , Hong CP , Oshima H , Oshima M . Gain‐of‐function p53 mutation acts as a genetic switch for TGFbeta signaling‐induced epithelial‐to‐mesenchymal transition in intestinal tumors. Cancer Res. 2024;84(1):56‐68. 37851521 10.1158/0008-5472.CAN-23-1490 PMC10758690

[189] Tian Y , Wang X , Cramer Z , et al. APC and P53 mutations synergise to create a therapeutic vulnerability to NOTUM inhibition in advanced colorectal cancer. Gut. 2023;72(12):2294‐2306. 37591698 10.1136/gutjnl-2022-329140 PMC10715527

[190] Luo Z , Wang B , Luo F , et al. Establishment of a large‐scale patient‐derived high‐risk colorectal adenoma organoid biobank for high‐throughput and high‐content drug screening. BMC Med. 2023;21(1):336. 37667332 10.1186/s12916-023-03034-y PMC10478412

[191] Ohta Y , Fujii M , Takahashi S , et al. Cell‐matrix interface regulates dormancy in human colon cancer stem cells. Nature. 2022;608(7924):784‐794. 35798028 10.1038/s41586-022-05043-y

[192] Zhong Q , Wang HG , Yang JH , et al. Loss of ATOH1 in pit cell drives stemness and progression of gastric adenocarcinoma by activating AKT/mTOR signaling through GAS1. Adv Sci (Weinh). 2023;10(32):e2301977. 37824217 10.1002/advs.202301977 PMC10646280

[193] Ye H , Shi W , Yang J , et al. PICH activates cyclin A1 transcription to drive S‐phase progression and chemoresistance in gastric cancer. Cancer Res. 2023;83(22):3767‐3782. 37646571 10.1158/0008-5472.CAN-23-1331

[194] Beekhof R , Bertotti A , Bottger F , et al. Phosphoproteomics of patient‐derived xenografts identifies targets and markers associated with sensitivity and resistance to EGFR blockade in colorectal cancer. Sci Transl Med. 2023;15(709):eabm3687. 37585503 10.1126/scitranslmed.abm3687

[195] Qiu J , Feng M , Yang G , et al. PRKRA promotes pancreatic cancer progression by upregulating MMP1 transcription via the NF‐kappaB pathway. Heliyon. 2023;9(6):e17194. 37484321 10.1016/j.heliyon.2023.e17194 PMC10361375

[196] Shimizu S , Kondo J , Onuma K , et al. Inhibition of the bone morphogenetic protein pathway suppresses tumor growth through downregulation of epidermal growth factor receptor in MEK/ERK‐dependent colorectal cancer. Cancer Sci. 2023;114(9):3636‐3648. 37357017 10.1111/cas.15882 PMC10475764

[197] Cioce M , Fumagalli MR , Donzelli S , et al. Interrogating colorectal cancer metastasis to liver: a search for clinically viable compounds and mechanistic insights in colorectal cancer patient derived organoids. J Exp Clin Cancer Res. 2023;42(1):170. 37460938 10.1186/s13046-023-02754-6 PMC10351152

[198] Morrison DD , Thompson DP , Semeyn DR , Bennett JL . Acute effects of oltipraz on adult Schistosoma mansoni and its antagonism in vitro. Biochem Pharmacol. 1987;36(7):1169‐1171. 3566810 10.1016/0006-2952(87)90429-1

[199] Wright CW , Li N , Shaffer L , et al. Establishment of a 96‐well transwell system using primary human gut organoids to capture multiple quantitative pathway readouts. Sci Rep. 2023;13(1):16357. 37773535 10.1038/s41598-023-43656-z PMC10541891

[200] Jensen LH , Rogatto SR , Lindebjerg J , et al. Precision medicine applied to metastatic colorectal cancer using tumor‐derived organoids and in‐vitro sensitivity testing: a phase 2, single‐center, open‐label, and non‐comparative study. J Exp Clin Cancer Res. 2023;42(1):115. 37143108 10.1186/s13046-023-02683-4 PMC10161587

[201] Dijkstra KK , van den Berg JG , Weeber F , et al. Patient‐derived organoid models of human neuroendocrine carcinoma. Front Endocrinol. 2021;12:627819. 10.3389/fendo.2021.627819 PMC7991829 33776923

[202] Dieterich W , Neurath MF , Zopf Y . Intestinal ex vivo organoid culture reveals altered programmed crypt stem cells in patients with celiac disease. Sci Rep. 2020;10(1):3535. 32103108 10.1038/s41598-020-60521-5 PMC7044285

[203] Meindl‐Beinker NM , Betge J , Gutting T , et al. A multicenter open‐label phase II trial to evaluate nivolumab and ipilimumab for 2nd line therapy in elderly patients with advanced esophageal squamous cell cancer (RAMONA). BMC Cancer. 2019;19(1):231. 30871493 10.1186/s12885-019-5446-2 PMC6419339

[204] van de Haar J , Ma X , Ooft SN , et al. Codon‐specific KRAS mutations predict survival benefit of trifluridine/tipiracil in metastatic colorectal cancer. Nat Med. 2023;29(3):605‐614. 36864254 10.1038/s41591-023-02240-8 PMC10033412

[205] Tian J , Chen JH , Chao SX , et al. Combined PD‐1, BRAF and MEK inhibition in BRAFV600E colorectal cancer: a phase 2 trial. Nat Med. 2023;29(2):458‐466. 36702949 10.1038/s41591-022-02181-8 PMC9941044

[206] Smyth EC , Vlachogiannis G , Hedayat S , et al. EGFR amplification and outcome in a randomised phase III trial of chemotherapy alone or chemotherapy plus panitumumab for advanced gastro‐oesophageal cancers. Gut. 2021;70(9):1632‐1641. 33199443 10.1136/gutjnl-2020-322658 PMC8355876

[207] Lie MRKL , van der Giessen J , Fuhler GM , et al. Low dose Naltrexone for induction of remission in inflammatory bowel disease patients. J Transl Med. 2018;16(1):55. 29523156 10.1186/s12967-018-1427-5 PMC5845217

[208] Reyes‐Uribe L , Wu W , Gelincik O , et al. Naproxen chemoprevention promotes immune activation in Lynch syndrome colorectal mucosa. Gut. 2021;70(3):555‐566. 32641470 10.1136/gutjnl-2020-320946 PMC7790993

[209] Taraborrelli L , Senbabaoglu Y , Wang L , et al. Tumor‐intrinsic expression of the autophagy gene Atg16l1 suppresses anti‐tumor immunity in colorectal cancer. Nat Commun. 2023;14(1):5945. 37741832 10.1038/s41467-023-41618-7 PMC10517947

[210] Lin W , Zhang Y , Yang Y , et al. Anti‐PD‐1/Her2 bispecific antibody IBI315 enhances the treatment effect of Her2‐positive gastric cancer through gasdermin B‐cleavage induced pyroptosis. Adv Sci (Weinh). 2023;10(30):e2303908. 37587833 10.1002/advs.202303908 PMC10602533

[211] Yao L , Hou J , Wu X , et al. Cancer‐associated fibroblasts impair the cytotoxic function of NK cells in gastric cancer by inducing ferroptosis via iron regulation. Redox Biol. 2023;67:102923. 37832398 10.1016/j.redox.2023.102923 PMC10582581

[212] Teijeira A , Migueliz I , Garasa S , et al. Three‐dimensional colon cancer organoids model the response to CEA‐CD3 T‐cell engagers. Theranostics. 2022;12(3):1373‐1387. 35154495 10.7150/thno.63359 PMC8771540

[213] Pickles OJ , Wanigasooriya K , Ptasinska A , et al. MHC class II is induced by IFNgamma and follows three distinct patterns of expression in colorectal cancer organoids. Cancer Res Commun. 2023;3(8):1501‐1513. 37565053 10.1158/2767-9764.CRC-23-0091 PMC10411481

[214] Potenza A , Balestrieri C , Spiga M , et al. Revealing and harnessing CD39 for the treatment of colorectal cancer and liver metastases by engineered T cells. Gut. 2023;72(10):1887‐1903. 37399271 10.1136/gutjnl-2022-328042

[215] Ding S , Hsu C , Wang Z , et al. Patient‐derived micro‐organospheres enable clinical precision oncology. Cell Stem Cell. 2022;29(6):905‐917. e6. 35508177 10.1016/j.stem.2022.04.006 PMC9177814

[216] Engevik MA , Danhof HA , Shrestha R , et al. Reuterin disrupts Clostridioides difficile metabolism and pathogenicity through reactive oxygen species generation. Gut Microbes. 2020;12(1):1788898. 10.1080/19490976.2020.1795388 PMC7524292 32804011

[217] Shao Y , Evers SS , Shin JH , et al. Vertical sleeve gastrectomy increases duodenal Lactobacillus spp. richness associated with the activation of intestinal HIF2alpha signaling and metabolic benefits. Mol Metab. 2022;57:101432. 34998940 10.1016/j.molmet.2022.101432 PMC8790500

[218] Deleu S , Arnauts K , Deprez L , et al. High acetate concentration protects intestinal barrier and exerts anti‐inflammatory effects in organoid‐derived epithelial monolayer cultures from patients with ulcerative colitis. Int J Mol Sci. 2023;24(1):768. 36614212 10.3390/ijms24010768 PMC9821118

[219] Mackie GM , Copland A , Takahashi M , et al. Bacterial cancer therapy in autochthonous colorectal cancer affects tumor growth and metabolic landscape. JCI Insight. 2021;6(23):e139900. 34710062 10.1172/jci.insight.139900 PMC8675204

[220] Green SI , Gu Liu C , Yu X , et al. Targeting of mammalian glycans enhances phage predation in the gastrointestinal tract. mBio. 2021;12(1):e03474. 33563833 10.1128/mBio.03474-20 PMC7885116

[221] Lavelle A , Sokol H . Gut microbiota‐derived metabolites as key actors in inflammatory bowel disease. Nat Rev Gastroenterol Hepatol. 2020;17(4):223‐237. 32076145 10.1038/s41575-019-0258-z

[222] Hanssen NMJ , de Vos WM , Nieuwdorp M . Fecal microbiota transplantation in human metabolic diseases: from a murky past to a bright future? Cell Metab. 2021;33(6):1098‐1110. 34077717 10.1016/j.cmet.2021.05.005

[223] Davoudi Z , Peroutka‐Bigus N , Bellaire B , Jergens A , Wannemuehler M , Wang Q . Gut organoid as a new platform to study alginate and chitosan mediated PLGA nanoparticles for drug delivery. Mar Drugs. 2021;19(5):282. 34065505 10.3390/md19050282 PMC8161322

[224] Cai T , Qi Y , Jergens A , Wannemuehler M , Barrett TA , Wang Q . Effects of six common dietary nutrients on murine intestinal organoid growth. PLoS One. 2018;13(2):e0191517. 29389993 10.1371/journal.pone.0191517 PMC5794098

[225] Peng H , Wang C , Xu X , Yu C , Wang Q . An intestinal Trojan horse for gene delivery. Nanoscale. 2015;7(10):4354‐4360. 25619169 10.1039/c4nr06377e

[226] Davoudi Z , Peroutka‐Bigus N , Bellaire B , et al. Intestinal organoids containing poly(lactic‐co‐glycolic acid) nanoparticles for the treatment of inflammatory bowel diseases. J Biomed Mater Res A. 2018;106(4):876‐886. 29226615 10.1002/jbm.a.36305 PMC5826879

[227] Tong T , Qi Y , Rollins D , et al. Rational design of oral drugs targeting mucosa delivery with gut organoid platforms. Bioact Mater. 2023;30:116‐128. 37560199 10.1016/j.bioactmat.2023.07.014 PMC10406959

[228] Khan R , Roy N , Ali H , Naeem M . Fecal microbiota transplants for inflammatory bowel disease treatment: synthetic‐ and engineered communities‐based microbiota transplants are the future. Gastroenterol Res Pract. 2022;2022:9999925. 35140783 10.1155/2022/9999925 PMC8820897

[229] Carberry BJ , Hergert JE , Yavitt FM , et al. 3D printing of sacrificial thioester elastomers using digital light processing for templating 3D organoid structures in soft biomatrices. Biofabrication. 2021;13(4). 10.1088/1758-5090/ac1c98 PMC8860055 34380115

[230] Kim S , Min S , Choi YS , et al. Tissue extracellular matrix hydrogels as alternatives to Matrigel for culturing gastrointestinal organoids. Nat Commun. 2022;13(1):1692. 35354790 10.1038/s41467-022-29279-4 PMC8967832

[231] Chakraborty J , Chawla S , Ghosh S . Developmental biology‐inspired tissue engineering by combining organoids and 3D bioprinting. Curr Opin Biotechnol. 2022;78:102832. 36332345 10.1016/j.copbio.2022.102832

[232] Zhang YS , Pi Q , van Genderen AM . Microfluidic bioprinting for engineering vascularized tissues and organoids. J Vis Exp. 2017;(126):55957. 28829418 10.3791/55957 PMC5614273

[233] Hendriks D , Clevers H , Artegiani B . CRISPR‐Cas tools and their application in genetic engineering of human stem cells and organoids. Cell Stem Cell. 2020;27(5):705‐731. 33157047 10.1016/j.stem.2020.10.014

[234] Artegiani B , Hendriks D , Beumer J , et al. Fast and efficient generation of knock‐in human organoids using homology‐independent CRISPR‐Cas9 precision genome editing. Nat Cell Biol. 2020;22(3):321‐331. 32123335 10.1038/s41556-020-0472-5

[235] Luna Velez MV , Neikes HK , Snabel RR , et al. ONECUT2 regulates RANKL‐dependent enterocyte and microfold cell differentiation in the small intestine; a multi‐omics study. Nucleic Acids Res. 2023;51(3):1277‐1296. 36625255 10.1093/nar/gkac1236 PMC9943655

[236] Lindeboom RG , van Voorthuijsen L , Oost KC , et al. Integrative multi‐omics analysis of intestinal organoid differentiation. Mol Syst Biol. 2018;14(6):e8227. 29945941 10.15252/msb.20188227 PMC6018986

[237] Qin Y , Palayyan SR , Zheng X , Tian S , Margolskee RF , Sukumaran SK . Type II taste cells participate in mucosal immune surveillance. PLoS Biol. 2023;21(1):e3001647. 36634039 10.1371/journal.pbio.3001647 PMC9836272

[238] Schuster B , Junkin M , Kashaf SS , et al. Automated microfluidic platform for dynamic and combinatorial drug screening of tumor organoids. Nat Commun. 2020;11(1):5271. 33077832 10.1038/s41467-020-19058-4 PMC7573629

[239] Sahoo D , Swanson L , Sayed IM , et al. Artificial intelligence guided discovery of a barrier‐protective therapy in inflammatory bowel disease. Nat Commun. 2021;12(1):4246. 34253728 10.1038/s41467-021-24470-5 PMC8275683

[240] Wani AK , Roy P , Kumar V , Mir TUG . Metagenomics and artificial intelligence in the context of human health. Infect Genet Evol. 2022;100:105267. 35278679 10.1016/j.meegid.2022.105267

[241] Seyed Tabib NS , Madgwick M , Sudhakar P , Verstockt B , Korcsmaros T , Vermeire S . Big data in IBD: big progress for clinical practice. Gut. 2020;69(8):1520‐1532. 32111636 10.1136/gutjnl-2019-320065 PMC7398484

